# Lysosomal TFEB‐TRPML1 Axis in Astrocytes Modulates Depressive‐like Behaviors

**DOI:** 10.1002/advs.202403389

**Published:** 2024-09-12

**Authors:** Jia‐Wen Mo, Peng‐Li Kong, Li Ding, Jun Fan, Jing Ren, Cheng‐Lin Lu, Fang Guo, Liang‐Yu Chen, Ran Mo, Qiu‐Ling Zhong, You‐Lu Wen, Ting‐Ting Gu, Qian‐Wen Wang, Shu‐Ji Li, Ting Guo, Tian‐Ming Gao, Xiong Cao

**Affiliations:** ^1^ Key Laboratory of Mental Health of the Ministry of Education Guangdong‐Hong Kong‐Macao Greater Bay Area Center for Brain Science and Brain‐Inspired Intelligence Guangdong‐Hong Kong Joint Laboratory for Psychiatric Disorders Guangdong Province Key Laboratory of Psychiatric Disorders Guangdong Basic Research Center of Excellence for Integrated Traditional and Western Medicine for Qingzhi Diseases Department of Neurobiology School of Basic Medical Sciences Southern Medical University Guangzhou 510515 China; ^2^ Microbiome Medicine Center Department of Laboratory Medicine Zhujiang Hospital Southern Medical University Guangzhou 510260 China; ^3^ Department of Psychology and Behavior Guangdong 999 Brain Hospital Institute for Brain Research and Rehabilitation South China Normal University Guangzhou 510515 China; ^4^ Department of Bioinformatics School of Basic Medical Sciences Southern Medical University Guangzhou 510515 China; ^5^ Department of Oncology Nanfang Hospital Southern Medical University Guangzhou 510515 China

**Keywords:** astrocytes, ATP, depressive‐like behaviors, lysosomes, mucolipin TRP channel 1, transcription factor EB

## Abstract

Lysosomes are important cellular structures for human health as centers for recycling, signaling, metabolism and stress adaptation. However, the potential role of lysosomes in stress‐related emotions has long been overlooked. Here, it is found that lysosomal morphology in astrocytes is altered in the medial prefrontal cortex (mPFC) of susceptible mice after chronic social defeat stress. A screen of lysosome‐related genes revealed that the expression of the mucolipin 1 gene (*Mcoln1*; protein: mucolipin TRP channel 1) is decreased in susceptible mice and depressed patients. Astrocyte‐specific knockout of mucolipin TRP channel 1 (TRPML1) induced depressive‐like behaviors by inhibiting lysosomal exocytosis‐mediated adenosine 5′‐triphosphate (ATP) release. Furthermore, this stress response of astrocytic lysosomes is mediated by the transcription factor EB (TFEB), and overexpression of TRPML1 rescued depressive‐like behaviors induced by astrocyte‐specific knockout of TFEB. Collectively, these findings reveal a lysosomal stress‐sensing signaling pathway contributing to the development of depression and identify the lysosome as a potential target organelle for antidepressants.

## Graphical abstract

This study indicates that chronic stress alters the morphology of astrocytic lysosomes and the mucolipin TRP channel 1 (TRPML1) expression in the mPFC of susceptible mice. Astrocyte‐specific knockout of TRPML1 results in depressive‐like behaviors in mice by inhibiting lysosomal exocytosis‐mediated adenosine 5'‐triphosphate (ATP) release. This stress response of astrocytic lysosomes is mediated by the transcription factor EB (TFEB).

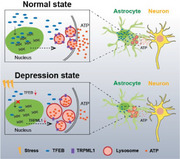

## 1 Introduction

The high lifetime risk (15%‐18%) and the leading cause of disability and suicide make major depressive disorder (MDD) one of the most common and devastating psychiatric illnesses worldwide.^[^ ^1^ ^]^ Susceptibility to depression is influenced by a variety of risk factors, among which stress is the most significant susceptibility factor.^[^ ^2^, ^3^, ^4^ ^]^ Many investigations have focused on the transmission of stress signals between brain cells, particularly astrocytes and neurons.^[^ ^5^, ^6^, ^7^, ^8^, ^9^ ^]^ In contrast, less attention has been given to the effects of depression‐related stress on intracellular organelles, and the mechanisms by which intracellular organelles affect the pathophysiology of depression remain largely unknown.

Lysosomes have long been regarded as the major degradation and recycling centers of the cell and more recently have been recognized as intracellular stress sensors and dynamic regulators of cellular and organismal homeostasis.^[^ ^10^, ^11^ ^]^ In the brain, lysosomes are involved in the regulation of synaptic plasticity, neuronal degeneration and death.^[^ ^12^, ^13^, ^14^, ^15^, ^16^, ^17^ ^]^ The impaired function of lysosomes underlies multiple lysosomal storage disorders (LSDs), and most LSDs have a progressive neurodegenerative or depression clinical course.^[^ ^18^, ^19^, ^20^ ^]^ Lysosomal dysfunction has been implicated in the onset of neurodegenerative diseases.^[^ ^14^ ^]^ However, the role of lysosomes in the pathogenesis of depression remains elusive.

Here, we detected abnormities in lysosomal morphology in medial prefrontal cortex (mPFC) astrocytes of susceptible mice after chronic social defeat stress (CSDS). A screen of lysosome‐related genes revealed that the levels of mucolipin 1 (*Mcoln1*) mRNA were decreased in susceptible mice and depressed patients. Astrocytic mucolipin TRP channel 1 (TRPML1) in the mPFC modulated depressive‐like behaviors in mice. The absence of TRPML1 in astrocytes suppressed lysosomal exocytosis, resulting in decreased ATP release in the mPFC. We further revealed that transcription factor EB (TFEB) mediated ATP release and depressive‐like behaviors in a TRPML1‐dependent manner.

## 2 Results

### 2.1 Lysosomal Morphology and TRPML1 Levels in mPFC Astrocytes are Altered Following CSDS

**Figure 1:**
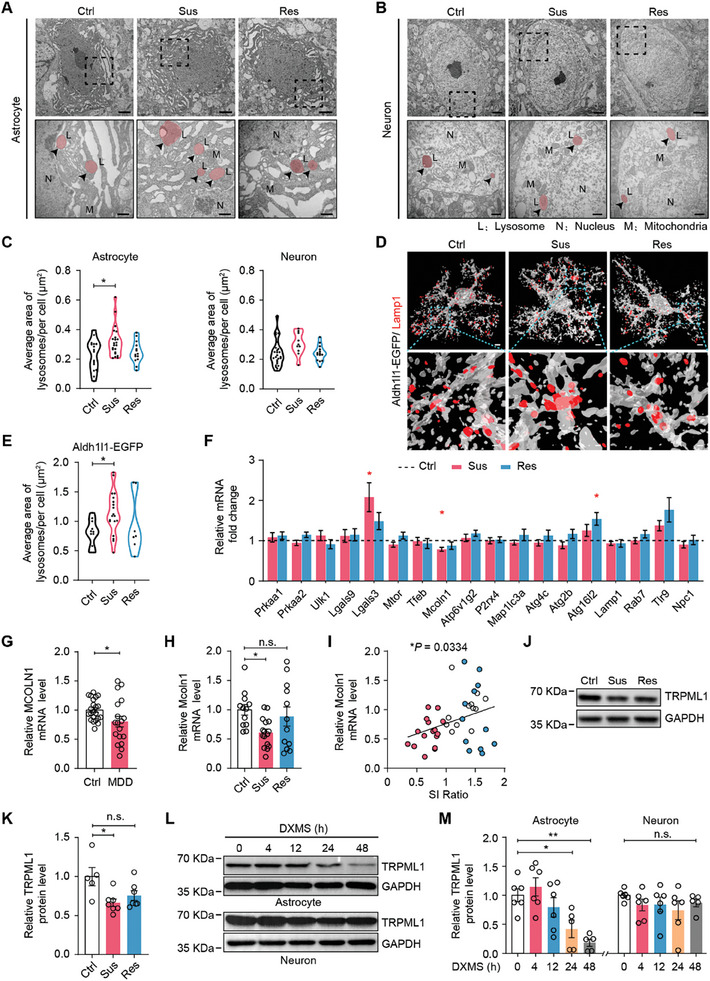
Lysosomal morphology and TRPML1 levels in mPFC astrocytes are altered following CSDS. A,B) Transmission electron microscopy images of lysosomes in mPFC astrocytes and neurons from Ctrl, Sus, and Res mice following the CSDS paradigm. The black arrows indicate lysosomes (red marks) in the cell. Scale bars, 2 µm (overview) and 500 nm (inset). C) Quantification of lysosomal size in the mPFC of Ctrl, Sus and Res mice (Astrocyte: n = 16 cells from 4 Ctrl mice, n = 22 cells from 6 Sus mice, n = 14 cells from 5 Res mice, *p* = 0.027; Neuron: n = 25 cells from 4 Ctrl mice, n = 10 cells from 6 Sus mice, n = 14 cells from 5 Res mice). D) Representative 3D‐reconstructed images of lysosomes immunostained with Lamp1 in mPFC astrocytes of *Aldh1l1‐EGFP* mice after the CSDS paradigm (EGFP, gray; Lamp1, red). Scale bars, 5 µm (overview) and 500 nm (inset). E) Quantification of lysosomal size in mPFC astrocytes from *Aldh1l1‐EGFP* mice after the CSDS paradigm (n = 11 cells from 3 Ctrl mice, n = 19 cells from 3 Sus mice, n = 7 cells from 3 Res mice, *p* = 0.0302). F) mRNA levels of lysosome‐related genes in the mPFC of Ctrl, Sus and Res mice (n = 9–10, each sample was analyzed in duplicate, *Lgals3: p* = 0.0143; *Mcoln1*: *p* = 0.0238; *Atg16l2: p* = 0.0206). G) *MCOLN1* mRNA levels in the peripheral blood of MDD patients and healthy controls (n = 18–22, each sample was analyzed in duplicate, *p* = 0.0341). H) *Mcoln1* mRNA levels in the peripheral blood of Ctrl, Sus and Res mice (n = 12–16, each sample was analyzed in duplicate, *p* = 0.015). I) Correlation between *Mcoln1* mRNA levels and SI ratio. *p* = 0.0334. J,K) Protein levels of TRPML1 in the mPFC of Ctrl, Sus and Res mice (n = 5–7, *p* = 0.0112). L,M) Western blotting analysis of TRPML1 protein levels in cultured astrocytes and neurons treated with DXMS (1 µM) for 0, 4, 12, 24, or 48 h (n = 5–6, *p* = 0.023 and *p* = 0.0017). One way ANOVA followed by Dunnett's post‐hot test or Kruskal‐Wallis test followed by Dunn's multiple comparisons test C,E,F,H,K and M); Mann Whitney test G) and correlations evaluated with the Pearson correlation coefficient I). All data are presented as the mean ± SEM. n.s., not significant; *p <0.05, **p <0.01.

To investigate whether lysosomes are involved in depression, we first employed the chronic social defeat stress (CSDS) model, a well‐established mouse model that mimics several psychopathological dimensions of depression.^[^ ^21^ ^]^ Adult male C57BL/6J mice were exposed to a larger and physically aggressive CD‐1 mouse (10 min/day) for 10 days (Figure S1A, Supporting Information). In the social interaction (SI) test, mice that displayed social avoidance were considered susceptible (Sus) mice, and mice that did not show social avoidance were considered resilient (Res) mice (Figure S1B, Supporting Information). Transmission electron microscopy was subsequently used to determine the number and morphology of lysosomes in the mPFC, a key functional hub involved in MDD.^[^ ^8^, ^22^, ^23^ ^]^ We imaged and analyzed lysosomes in astrocytes and neurons, two major cell types in the brain.^[^ ^24^ ^]^ Quantitative analysis revealed that the number and size of astrocytic lysosomes were increased in Sus mice, compared to Ctrl mice (**Figure** **1**A,C; Figure S1C, Supporting Information). However, no significant changes in lysosomal morphology were observed in neurons of Sus and Res mice (Figure 1B,C; Figure S1D, Supporting Information). Furthermore, lysosomes were labeled with Lamp1, a typical marker for lysosomal membranes, and imaged via Structure Illumination Microscopy (SIM). 3D‐reconstructed SIM images also revealed that the size of lysosomes was significantly increased in mPFC astrocytes of Sus mice, compared to Ctrl mice (Figure 1D,E; Figure S1E, Supporting Information). Meanwhile, Lamp1 protein levels were increased in the mPFC of Sus mice (Figure S1F, Supporting Information).

To identify lysosome‐related components involved in depression, we next used the quantitative real‐time PCR (qRT‐PCR) to examine the expression of a panel of genes related to the regulation of lysosomal processes in the mPFC. Quantitative analysis revealed that *Mcoln1* mRNA levels were decreased in Sus mice and that *Lgals3* levels were upregulated in Sus mice compared to Ctrl mice. *Atg16l2* levels were significantly increased in Res mice, and no obvious changes in the expression of other genes were observed (Figure 1F). Subsequently, we examined the mRNA levels of *MCOLN1* and *LGALS3* in the peripheral blood of MDD patients and healthy control subjects. Compared to healthy controls, MDD patients showed decreased *MCOLN1* mRNA expression in the peripheral blood, which was consistent with the change in *Mcoln1* mRNA expression in the mPFC of Sus mice (Figure 1F,G). *LGALS3* levels were attenuated in the peripheral blood of MDD patients, in contrast to the change in *Lgals3* expression in the mPFC of Sus mice (Figure 1F; Figure S1G, Supporting Information). In addition, *Mcoln1* mRNA levels in the peripheral blood of Sus mice were also significantly reduced (Figure 1H), and *Mcoln1* levels were positively correlated with the SI ratio (Figure 1I). The *Mcoln1* gene encodes the protein TRPML1, which is the principal Ca^2+^ channel in the lysosome.^[^ ^25^ ^]^ Western blotting confirmed that TRPML1 protein levels were reduced in the mPFC of Sus mice (Figure 1J,K), and TRPML1 levels were positively correlated with the SI ratio (Figure S1H, Supporting Information).

To gain insight into the changes in TRPML1 expression in brain cells, we measured TRPML1 protein levels in cultured astrocytes, neurons, and microglial BV2 cells treated with dexamethasone (DXMS), which mimics the depression‐related stress response in vitro.^[^ ^23^ ^]^ Western blotting revealed that TRPML1 protein levels were significantly decreased in cultured astrocytes treated with DXMS (1 µM) for 24 and 48 h, but no differences were observed in neurons or microglial BV2 cells (Figure 1L,M; Figure S2A, Supporting Information). We then isolated astrocytes from the mPFC by fluorescence‐activated cell sorting (FACS) (Figure S1I, Supporting Information). Simple Western blotting showed that astrocytic TRPML1 expression was significantly decreased in the mPFC after the CSDS paradigm (Figure S1J, Supporting Information). The fluorescence immunostaining also revealed lower intensity of TRPML1 in S100β^+^ astrocytes in the mPFC from C57BL/6J mice after the CSDS paradigm (Figure S1K, Supporting Information), while chronic social stress did not affect TRPML1 levels in mPFC Iba1^+^ microglia of Sus mice (Figure S2B, Supporting Information). Taken together, these results suggest that lysosomal TRPML1 in astrocytes may be involved in chronic stress and consequent depressive‐like phenotypes.

### 2.2 Astrocyte‐Specific Knockout of TRPML1 Results in Depressive‐Like Behaviors in Mice

**Figure 2:**
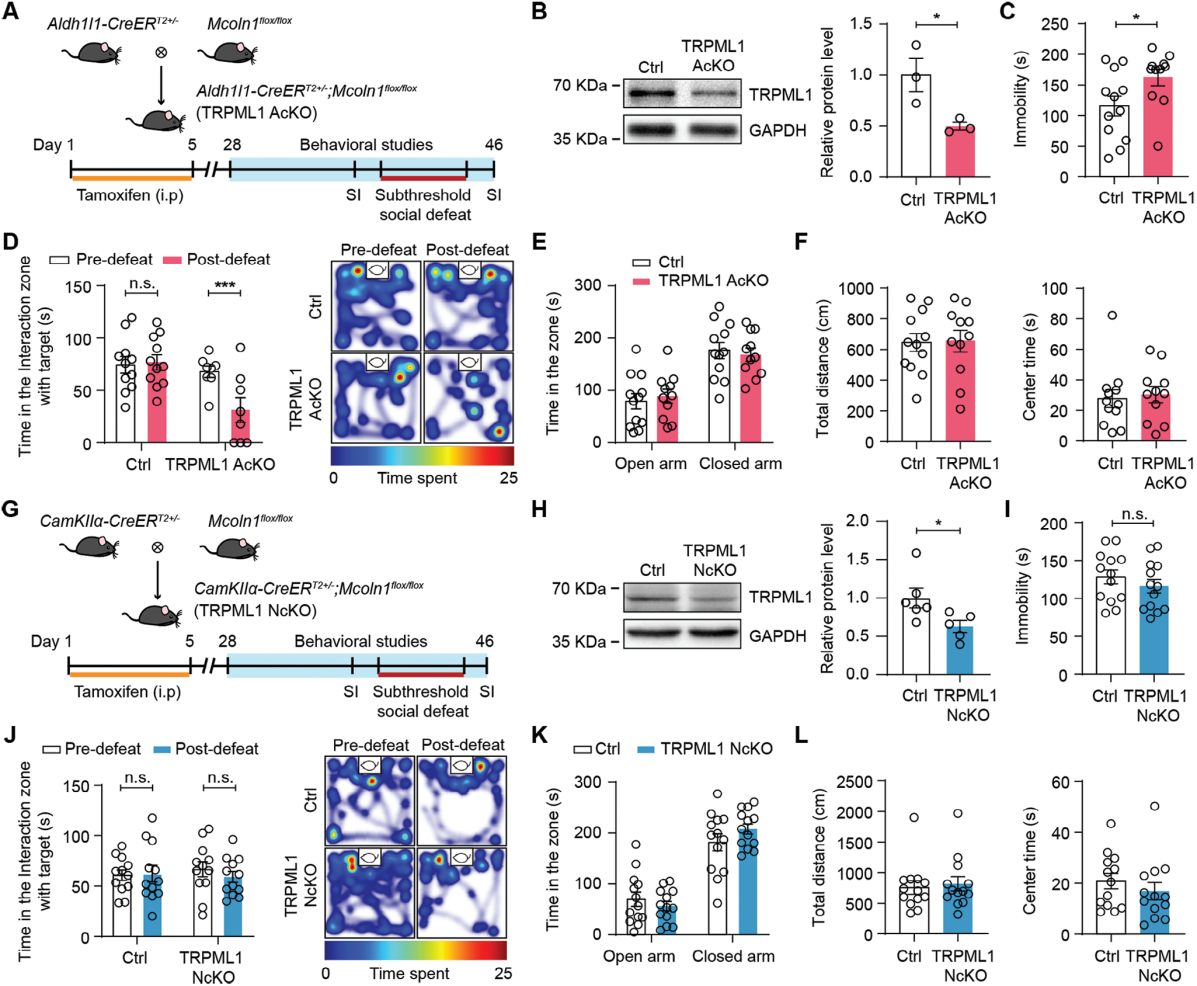
Selective deletion of astrocytic *Mcoln1* induces depressive‐like behaviors in mice. A) Generation of astrocyte‐specific TRPML1 knockout mice (TRPML1 AcKO) and experimental timeline. B) Western blotting analysis of TRPML1 expression in the mPFC of TRPML1 AcKO and Ctrl mice (n = 3, *p =* 0.0425). C–F) Behavioral performances of TRPML1 AcKO and Ctrl mice in FST C) (n = 11–12, *p* = 0.0383), SI test D) (n = 8–11, *p* = 0.0009), EPM E) and OFT F) (n = 11–12). G) Generation of neuron‐specific TRPML1 knockout mice (TRPML1 NcKO) and experimental timeline. H) Western blotting analysis of TRPML1 expression in the mPFC of TRPML1 NcKO and Ctrl mice (n = 5–6, *p* = 0.043). I–L) Behavioral performances of TRPML1 NcKO and Ctrl mice in FST I) (n = 13), SI test J) (n = 12), EPM K), and OFT L) (n = 13). Two‐tailed unpaired Student's t test B,C,E,F,H,I,K and L); two‐way ANOVA followed by Sidak's multiple comparisons test D and J). All data are presented as the mean ± SEM. n.s., not significant; *p <0.05, ***p <0.001.

To determine whether astrocytic TRPML1 is critical for depressive‐like behaviors, we generated astrocyte‐specific *Mcoln1* deletion mouse lines (TRPML1 AcKO) by crossing mice expressing the floxed *Mcoln1* allele with *Aldh1l1‐CreER^T2+/‐^* mice, which express Cre recombinase specifically in astrocytes when treated with tamoxifen (**Figure** **2**A).^[^ ^26^ ^]^ Western blotting showed that TRPML1 protein levels were significantly decreased in the mPFC of TRPML1 AcKO mice, compared to Ctrl mice (Figure 2B). To further confirm the astrocyte‐specific TRPML1 reduction in TRPML1 AcKO animals, astrocytes were isolated from the mPFC by FACS, and Simple Western blot analysis showed that TRPML1 protein levels were decreased in mPFC astrocytes of TRPML1 AcKO mice (Figure S3B, Supporting Information). TRPML1 AcKO mice did not exhibit changes in body weight, brain size or structure, astrocyte and neuron density compared to littermate controls (Figure S3C–G, Supporting Information).

We then assessed the behavioral performances of TRPML1 AcKO mice. In the forced swimming test (FST), TRPML1 AcKO mice exhibited a significant increase in immobility duration (Figure 2C). Next, TRPML1 AcKO mice were subjected to a subthreshold social defeat stress (SSDS) experiment and performed to the SI test before and after SSDS to assess depression‐related stress vulnerability.^[^ ^23^, ^27^ ^]^ We found that Ctrl mice spent a similar amount of time in the interaction zone after 3 days of SSDS as before SSDS. However, TRPML1 AcKO mice exhibited a reduced retention time spent in the interaction zone after SSDS, indicating greater social avoidance than before SSDS (Figure 2D). TRPML1 AcKO mice also showed less preference of sucrose in the sucrose preference test, compared to Ctrl mice (Figure S3H, Supporting Information). No obvious differences in anxiety‐like behavior in the elevated plus mazes (EPM) test, general locomotion in the open field test (OFT), and motor coordination in rotarod and pole climbing tests were observed between TRPML1 AcKO and Ctrl mice (Figure 2E,F; Figure S3I,J, Supporting Information).

In addition, we generated a neuron‐specific *Mcoln1* deletion mouse line (TRPML1 NcKO) by crossing mice expressing the floxed *Mcoln1* allele with *CamKIIa‐CreER^T2+/−^* mice (Figure 2G). Western blotting revealed that TRPML1 protein levels were significantly decreased in the mPFC of TRPML1 NcKO mice, compared to Ctrl mice (Figure 2H). To further confirm the neuron‐specific TRPML1 reduction in TRPML1 NcKO animals, neurons were isolated from the mPFC by magnetic‐activated cell sorting (MACS), and qPCR analysis showed that *Mcoln1* levels were significantly decreased in mPFC neurons of TRPML1 NcKO mice (Figure S4A, Supporting Information). TRPML1 NcKO mice did not show changes in body weight, brain size and structure, astrocyte and neuron density compared to littermate controls (Figure S4B–F, Supporting Information). In addition, TRPML1 NcKO mice did not display depressive‐like phenotypes in the FST (Figure 2I) and the SI tests performed before and after SSDS (Figure 2J). No differences were observed in the EPM test (Figure 2K) and OFT (Figure 2L). Taken together, these results suggest that TRPML1 deficiency in astrocytes promotes depressive‐like behaviors in mice.

### 2.3 Astrocytic TRPML1 in the mPFC Modulates Depressive‐Like Behaviors

**Figure 3:**
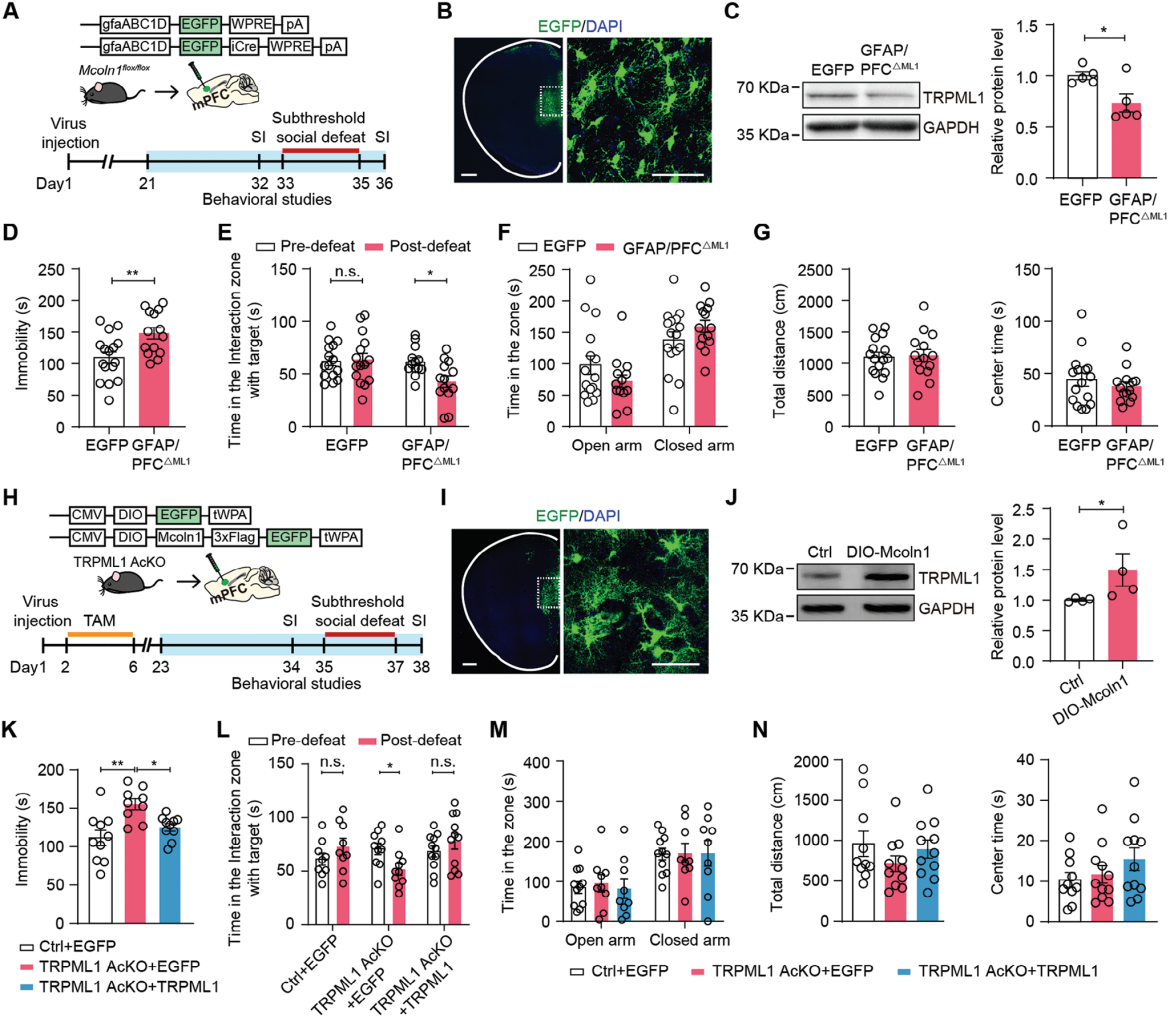
Astrocytic TRPML1 in the mPFC modulates depressive‐like behaviors. A) Schematic of the AAV vectors and design of the studies performed to analyze the behavioral performances of *Mcoln1^flox/flox^* mice infected with AAV‐gfaABC1D‐EGFP‐iCre or AAV‐gfaABC1D‐EGFP virus. B) Representative confocal images of AAV‐gfaABC1D‐EGFP‐iCre expression in astrocytes of the mPFC. Scale bars = 500 µm (left), 50 µm (right). C) Western blotting images and quantitative analysis of TRPML1 expression in the mPFC of GFAP/PFC^△ML1^ and EGFP mice (n = 5, *p* = 0.025). D–G) Behavioral performances of GFAP/PFC^△ML1^ and EGFP mice in FST D) (n = 14–16, *p* = 0.0058), SI test E) (n = 13–15, *p* = 0.0105), EPM F) and OFT G) (n = 14–16). H) Schematic of the AAV vectors and design of the studies performed to analyze the behavioral performances of TRPML1 AcKO mice infected with AAV‐DIO‐Mcoln1‐3×Flag‐EGFP or AAV‐DIO‐EGFP virus. I) Representative confocal images of AAV‐DIO‐Mcoln1‐3×Flag‐EGFP expression in mPFC astrocytes. Scale bars = 500 µm (left), 50 µm (right). J) Western blotting images and quantitative analysis of TRPML1 expression in the mPFC of TRPML1 AcKO mice infected with AAV‐DIO‐Mcoln1‐3×Flag‐EGFP or AAV‐DIO‐EGFP virus (n = 4, *p* = 0.0286). K–N) Behavioral performances of TRPML1 AcKO mice infected with AAV‐DIO‐Mcoln1‐3×Flag‐EGFP or AAV‐DIO‐EGFP virus in FST K) (n = 9–10, *p* = 0.0013, *p* = 0.0283), SI test L) (n = 9–11, *p* = 0.0207), EPM M), and OFT N) (n = 9–11). Two‐tailed unpaired Student's t test C,D,F,G); Mann Whitney test J); one way ANOVA followed by Tukey's multiple comparisons test K,M and N); two‐way ANOVA followed by Sidak's multiple comparisons test E and L). All data are presented as the mean ± SEM. n.s., not significant; *p <0.05, **p <0.01.

To determine the brain region specific effect of TRPML1 on behaviors, we bilaterally injected AAV‐gfaABC1D‐EGFP‐iCre or AAV‐gfaABC1D‐EGFP virus, which induces the expression of Cre recombinase or EGFP in astrocytes under the control of the human GFAP promoter,^[^ ^6^ ^]^ into the mPFC of *Mcoln1^flox/flox^* mice to obtain mPFC astrocyte‐specific TRPML1 knockout mice (GFAP/PFC^△ML1^) or control mice (EGFP) (**Figure** **3**A). Three weeks after injection, astrocytes throughout the mPFC were infected with the gfaABC1D‐iCre virus (Figure 3B). Western blotting analysis showed a decrease in TRPML1 expression in the mPFC after gfaABC1D‐iCre virus injection (Figure 3C). To further confirm the astrocyte‐specific TRPML1 reduction in GFAP/PFC^△ML1^ mice, astrocytes were isolated from the mPFC by MACS, and qPCR analysis showed that *Mcoln1* mRNA levels were decreased in mPFC astrocytes of GFAP/PFC^△ML1^ mice compared to EGFP mice (Figure S5A, Supporting Information). Next, we performed behavioral tests. In the FST, GFAP/PFC^△ML1^ mice showed increased immobility time compared to EGFP mice (Figure 3D). In the SI test, GFAP/PFC^△ML1^ mice spent less time in the interaction zone after 3 days of SSDS than before SSDS, indicating social avoidance (Figure 3E). In the EPM and OFT, no obvious differences in behaviors were observed between GFAP/PFC^△ML1^ and EGFP mice (Figure 3F,G).

To investigate whether restoration of astrocytic TRPML1 expression in the mPFC could reverse depressive‐like behaviors in TRPML1 AcKO mice, we bilaterally injected AAV‐DIO‐Mcoln1‐3×Flag‐EGFP or AAV‐DIO‐EGFP virus into the mPFC of TRPML1 AcKO and Ctrl mice (Figure 3H). Confocal images showed that astrocytes in the mPFC were infected with the virus (Figure 3I). Western blotting analysis showed an increase in TRPML1 protein levels in the mPFC of TRPML1 AcKO mice infected with DIO‐Mcoln1‐EGFP virus (Figure 3J). To further confirm the astrocyte‐specific TRPML1 restoration in TRPML1 AcKO mice, astrocytes were isolated from the mPFC by MACS. We found that *Mcoln1* mRNA levels were significantly increased in mPFC astrocytes of TRPML1 AcKO mice infected with DIO‐Mcoln1‐EGFP virus (Figure S5B, Supporting Information). In the behavioral tests, TRPML1 AcKO mice infected with the DIO‐EGFP virus also displayed depressive‐like phenotypes, including increased immobility time in the FST (Figure 3K), and social avoidance behavior in the SI test after SSDS (Figure 3L). These depressive‐like behaviors in TRPML1 AcKO mice were reversed by the restoration of TRPML1 expression in mPFC astrocytes (Figure 3K,L). No behavioral changes were observed in the EPM and OFT (Figure 3M,N). Taken together, these results suggest that TRPML1 deficiency in mPFC astrocytes is sufficient to induce depressive‐like behaviors in mice.

### 2.4 Knockdown of Astrocytic TRPML1 Impairs Lysosomal Exocytosis‐Mediated ATP Release in the mPFC

**Figure 4:**
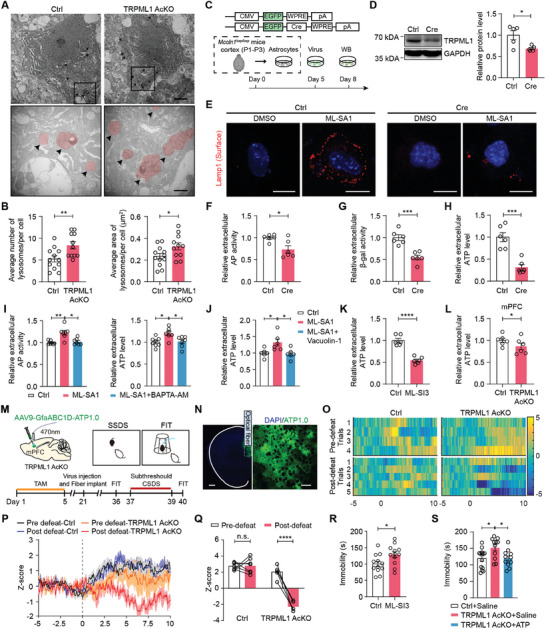
Knockdown of astrocytic TRPML1 decreases lysosomal exocytosis‐mediated ATP release in the mPFC. A,B) TEM images A) and quantitative analysis B) of lysosomes in mPFC astrocytes from TRPML1 AcKO and Ctrl mice (n = 11 cells from 2 TRPML1 AcKO mice, n = 12 cells from 2 Ctrl mice, *p* = 0.0089 and *p* = 0.0391). The black arrows indicate lysosomes (red marks) in the cell. Scale bars, 2 µm (overview) and 500 nm (inset). C) Design of the procedure for knockdown of astrocytic TRPML1 in vitro. D) Western blotting analysis of TRPML1 expression in cultured *Mcoln1^flox/flox^* astrocytes after infection with the Cre virus (n = 5, *p* = 0.0218). E) Lamp1 surface immunostaining in non‐permeabilized astrocytes. Scale bars, 10 µm. F‐H) Measurements of AP, β‐Gal activity and ATP levels in the medium of primary cultured astrocytes (n = 6). *p* = 0.0131 F), *p* = 0.0002 G) and *p* = 0.0002 H). I) Measurements of AP activity and ATP concentrations in the medium of primary cultured astrocytes treated with ML‐SA1 (50 µM, 1 h) and BAPTA‐AM (500 nM, 20 min) (n = 7–8). *p* = 0.0096 and *p* = 0.0119 (left), *p* = 0.0104 and *p* = 0.0199 (right). J) Measurements of ATP concentrations in the medium of primary cultured astrocytes treated with ML‐SA1 and Vacuolin‐1 (10 µM, 1 h) (n = 6, *p* = 0.0249 and *p* = 0.0161). K) Measurements of ATP concentrations in the medium of primary cultured astrocytes treated with ML‐SI3 (25 µM, 10 min) (n = 6, *p* <0.0001). L) ATP levels in the ACSF of isolated mPFC slices from TRPML1 AcKO mice (n = 6, *p* = 0.0313). M) Schematic of the AAV vectors engineered to express the ATP sensor and design of the fiber photometry experiment. N) Representative images of ATP sensor expression in mPFC astrocytes. Scale bars = 500 µm (left), 50 µm (right). O) Representative heat maps of z‐score changes over all trials in individual mice. P,Q) Time course of the average ATP transient z‐score event in the forced interaction test (FIT) (p) and quantification of the average peak z score during the FIT (Q) (n = 5–7, *p* <0.0001). R) Behavioral performances of C57BL/6J mice in the FST after infusion of ML‐SI3 into the mPFC (n = 12, *p* = 0.0281). S) Behavioral performances of TRPML1 AcKO mice treated with ATP in FST (n = 11–14, *p* = 0.0229 and *p* = 0.0363). Two‐tailed unpaired Student's t test B,D,F,G,H,K and R); Wilcoxon matched‐pairs signed rank test L); one way ANOVA followed by Tukey's multiple comparisons test I,J and S); two‐way ANOVA followed by Sidak's multiple comparisons test Q). All data are presented as the mean ± SEM. n.s., not significant; *p <0.05, **p <0.01, ***p <0.001, ****p <0.0001.

As the principal Ca^2+^ release channel in the lysosomes, TRPML1 plays a key role in most lysosomal trafficking processes.^[^ ^25^ ^]^ Several findings indicate that TRPML1 regulates lysosomal exocytosis by mediating Ca^2+^‐dependent membrane fusion and fission.^[^ ^28^, ^29^ ^]^ To investigate how lysosomal TRPML1 in mPFC astrocytes modulates depressive‐like behaviors, we first examined lysosomal morphology using transmission electron microscopy (**Figure** **4**A). Compared to those in Ctrl mice, the number and size of lysosomes were increased in mPFC astrocytes of TRPML1 AcKO mice (Figure 4B). Therefore, we next monitored the dynamics of lysosomal exocytosis in astrocytes in vitro. Primary cultured astrocytes from *Mcoln1^flox/flox^* mice (P1‐3) were infected with pLenti‐EGFP‐Cre (Cre) or pLenti‐EGFP (Ctrl) virus (Figure 4C). Western blotting analysis revealed that the TRPML1 protein levels were significantly reduced in astrocytes infected with the pLenti‐EGFP‐Cre virus (Figure 4D). We then activated TRPML1 using ML‐SA1, an agonist of TRPML1, and evaluated lysosomal exocytosis using Lamp1 surface immunostaining.^[^ ^30^ ^]^ Ctrl virus‐infected, but not Cre virus‐infected, astrocytes presented a marked increase in Lamp1 staining after treatment with the ML‐SA1 (Figure 4E). Meanwhile, astrocytic lysosomes were labeled with FM2‐10, an FM dye that can selectively accumulate in lysosomes, and then treated with glutamate to induce lysosomal exocytosis.^[^ ^31^ ^]^ Confocal time‐lapse imaging showed that the application of glutamate (1 mM) induced a 50% decrease in fluorescence intensity in Ctrl virus‐infected astrocytes but not in Cre virus‐infected astrocytes (Figure S6A, Supporting Information). These results suggest that the knockdown of astrocytic TRPML1 leads to dysfunction of lysosomal exocytosis.

Lysosomes are able to release a variety of substances by exocytosis, including hydrolases and transmitters such as adenosine 5′‐triphosphate (ATP).^[^ ^28^, ^31^, ^32^ ^]^ In astrocytes, lysosomal exocytosis plays a vital role in the release of ATP,^[^ ^31^ ^]^ which is a critical gliotransmitter that regulates depressive symptoms and antidepressant‐like effects in mice.^[^ ^22^, ^23^, ^33^, ^34^ ^]^ To determine whether astrocytic TRPML1 modulates lysosomal exocytosis‐mediated ATP release, we measured the levels of ATP and the activity of lysosomal hydrolytic enzymes such as acid phosphatases (AP) and β‐galactosidases (β‐Gal) in the extracellular medium.^[^ ^31^, ^35^ ^]^ The results showed that the knockdown of astrocytic TRPML1 significantly decreased extracellular AP and β‐Gal activity and ATP levels (Figure 4F–H). Previous studies have reported that TRPML1‐mediated lysosomal Ca^2+^ release triggers lysosomal exocytosis.^[^ ^28^, ^36^ ^]^ Therefore, we used a membrane‐permeable and fast Ca^2+^ chelator, BAPTA‐AM, to buffer Ca^2+^,^[^ ^37^ ^]^ and found that incubation with ML‐SA1 (50 µM) induced a significant increase in the AP and ATP release, which was abolished by BAPTA‐AM (500 nM) (Figure 4I). Furthermore, treatment with Vacuolin‐1 (10 µM), a potent and cell‐permeable inhibitor of lysosomal exocytosis,^[^ ^38^ ^]^ blocked ML‐SA1‐induced ATP release (Figure 4J). Meanwhile, we found that treatment with ML‐SI3, an antagonist of TRPML1,^[^ ^39^ ^]^ decreased ATP levels in the medium of primary astrocytes (Figure 4K). Notably, ATP concentrations in the artificial cerebrospinal fluid (ACSF) of mPFC slices isolated from TRPML1 AcKO mice were lower than those isolated from Ctrl mice (Figure 4L). Moreover, we used recorded extracellular ATP dynamics in the mPFC of mice subjected to the forced interaction test (FIT) by fiber photometry. An ATP sensor with high specificity and sensitivity, AAV‐GfaABC1D‐ATP1.0,^[^ ^40^ ^]^ was injected into the mPFC of TRPML1 AcKO and control littermates, and an optical fiber was then implanted above the infected cells (Figure 4M). Two weeks after virus injection, fluorescence signals from astrocytes were recorded while the mice were attacked by an aggressor mouse during the FIT (Figure 4N). Before the SSDS, there was no significant difference in the fluorescence intensity between TRPML1 AcKO and Ctrl mice during attacks by a CD‐1 aggressor mouse, as the fluorescence intensity was increased in both types of mice (Figure 4O,P). After the SSDS, Ctrl mice also showed a large increase in fluorescence intensity during attack periods. However, TRPML1 AcKO mice showed a marked decrease in fluorescence intensity during attack periods (Figure 4P,Q). These results indicate that TRPML1 deficiency results in the impairment of lysosomal exocytosis‐mediated ATP release in astrocytes.

Next, we employed cannula infusion of ML‐SI3 into the mPFC and found that the application of ML‐SI3 (25 µM) to the mPFC resulted in a significant increase in the immobility duration of C57BL/6J mice during the FST (Figure 4R), without behavioral changes in the OFT (Figure S6B, Supporting Information). To determine whether the depressive‐like behaviors induced by selective deletion of astrocytic *Mcoln1* could be rescued by the application of ATP, we treated TRPML1 AcKO mice with ATP (125 mg kg^−1^, i.p.) or saline. Compared to saline, ATP treatment reversed the increase in immobility time in TRPML1 AcKO mice in the FST (Figure 4S). No significant difference was observed in general locomotion in the OFT (Figure S6C, Supporting Information). Taken together, these results demonstrate that lysosomal exocytosis‐mediated ATP release in astrocytes is disrupted by TRPML1 deficiency, contributing to the depressive‐like behaviors.

Our previous studies show that astrocyte‐derived ATP regulates depressive‐like behaviors by modulating neuronal activity.^[^ ^22^, ^41^ ^]^ To investigate whether astrocytic TRPML1 is involved in regulating neuronal activity under stress in vivo, the Ca^2+^ indicator, GCaMP6s, under a *CamKIIa* promoter was injected into the mPFC of TRPML1 AcKO and Ctrl mice, and fiber photometry was used to record Ca^2+^ signals in mPFC neurons (Figure S7A and B, Supporting Information). Before the SSDS, both TRPML1 AcKO and Ctrl mice exhibited the same stimulus‐evoked intracellular Ca^2+^ elevations during attacks by a CD‐1 aggressor (Figure S7C and D, Supporting Information), and no significant difference in the fluorescence intensity was observed between the two groups of mice (Figure S7D and E, Supporting Information). After the SSDS, Ctrl mice also showed a large increase in fluorescence intensity during attack periods. However, TRPML1 AcKO mice showed a significant decrease in Ca^2+^ activity during the attack periods compared to before the SSDS (Figure S7D and E, Supporting Information). These results suggest that astrocytic TRPML1 deficiency leads to deficits in neuronal calcium activity in the mPFC under stress.

### 2.5 Astrocytic TFEB Mediates ATP Release in a TRPML1‐Dependent Manner

**Figure 5:**
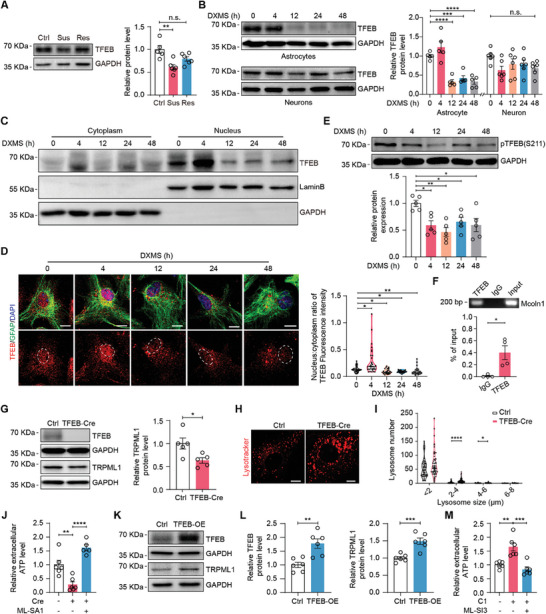
Astrocytic TFEB mediates ATP release in a TRPML1‐dependent manner. A) TFEB protein levels in the mPFC of Ctrl, Sus and Res mice (n = 5–6, *p* = 0.0014). B) Western blotting analysis of TFEB protein levels in cultured astrocytes and neurons treated with DXMS (1 µM) for 0, 4, 12, 24, or 48 h (n = 5–6, *p* <0.0001, *p* = 0.0005 and *p* <0.0001). C) Western blotting analysis of astrocytic TFEB levels in the nuclear and cytosolic fractions. D) Representative immunofluorescence images and quantification of TFEB nuclear translocation in cultured astrocytes treated with DXMS (n = 26–34 cells, *p* = 0.0248, *p* = 0.0287, *p* = 0.0494 and *p* = 0.0077). TFEB, red; GFAP, green; DAPI, blue. Scale bars, 10 µm. E) Western blotting analysis of pTFEB (Ser 211) levels in cultured astrocytes treated with DXMS (n = 5, *p* = 0.0125, *p* = 0.0012, *p* = 0.0387 and *p* = 0.0131). F) ChIP‐PCR assay using primary cultured astrocytes (n = 4, *p* = 0.0286). G) Western blotting analysis of TFEB and TRPML1 protein levels in cultured *Tfeb^flox/flox^* astrocytes infected with the Cre virus (n = 5, *p* = 0.0248). H) Representative images of Lysotracker staining. Scale bars, 5 µm. I) Quantification of lysosomal number in TFEB knockdown astrocytes (n = 44–48 cells, *p* <0.0001 and *p* = 0.0435). J) ATP levels in the medium of TFEB knockdown astrocytes treated with the TRPML1 agonist ML‐SA1 (50 µM) or vehicle for 1 h (n = 5–6, *p* = 0.0034 and *P* <0.0001). K,L) Western blotting analysis of TFEB and TRPML1 protein levels in 293T cells transfected with the TFEB plasmid (n = 6, *p* = 0.0028 and *p* = 0.0008). M) ATP levels in the medium of primary cultured astrocytes treated with the TFEB agonist C1 (1 µM, 12 h) and the TRPML1 antagonist ML‐SI3 (n = 6, *p* = 0.0013 and *p* = 0.0002). One way ANOVA followed by Dunnett's post‐hot test A,B and E); Kruskal‐Wallis test followed by Dunn's multiple comparisons test D); two‐tailed unpaired Student's t test G and L) and Mann Whitney test F and I); one way ANOVA followed by Tukey's multiple comparisons test J and M). All data are presented as the mean ± SEM. n.s., not significant; *p <0.05, **p <0.01, ***p <0.001, ****p <0.0001.

How do astrocytic lysosomes respond to depression‐related stress and regulate TRPML1‐dependent ATP release? TFEB is an important transcription factor that regulates the lysosomal response to cellular stimuli and modulates lysosome‐related genes, including TRPML1.^[^ ^25^, ^42^ ^]^ Previous studies have reported that TFEB regulates lysosomal exocytosis through TRPML1.^[^ ^28^ ^]^ To determine whether TFEB is involved in depression‐related stress, we first examined TFEB protein levels in the mPFC of C57BL/6J mice after the CSDS paradigm using Western blotting. TFEB protein levels were significantly attenuated in the mPFC of Sus mice compared to Ctrl mice (**Figure** **5**A). Furthermore, the TFEB protein level was positively correlated with the SI ratio (Figure S8A, Supporting Information). To test whether neurons or astrocytes contribute to the reduction in TFEB levels, primary cultured astrocytes and neurons were treated with DXMS (1 µM). The results showed that TFEB protein levels were downregulated in astrocytes exposed to DXMS for 12, 24 and 48 h, but no differences in TFEB protein levels were observed in neurons (Figure 5B). Simple Western blotting analysis also showed that TFEB levels were decreased in astrocytes isolated from the mPFC of Sus mice compared to those from Ctrl mice (Figure S8B,C, Supporting Information). Under stress conditions, TFEB is activated after dephosphorylation and translocates from the cytoplasm to the nucleus, thereby regulating the expression of lysosome‐related genes.^[^ ^42^, ^43^ ^]^ In DXMS time‐course experiments, 4 h of DXMS treatment induced the nuclear localization of TFEB and decreased the phosphorylation level of the serine residue Ser211 of TFEB, suggesting a normal response to stress (Figure 5C–E). However, DXMS treatment for ≥12 h inhibited the nuclear localization of TFEB and decreased the protein level of pTFEB (Ser211) (Figure 5C–E), consistent with the changes in total TFEB protein levels (Figure 5B).

We next examined whether TFEB regulates TRPML1 expression in astrocytes. First, the mRNA levels of several target genes bearing the coordinated lysosomal expression and regulation (CLEAR) motif under the control of TFEB were detected in primary cultured *Tfeb^flox/flox^* astrocytes infected with pLenti‐EGFP‐Cre or pLenti‐EGFP virus. We found that *Mcoln1* mRNA levels were significantly decreased in astrocytes infected with the pLenti‐EGFP‐Cre virus compared to astrocytes infected with the pLenti‐EGFP virus (Figure S8D, Supporting Information). Next, the chromatin immunoprecipitation‐PCR (ChIP‐PCR) assay revealed that TFEB binds to a specific motif within the *Mcoln1* promoter in astrocytes (Figure 5F). Western blotting analysis showed that the protein levels of TRPML1 were significantly reduced in Cre virus‐infected astrocytes (Figure 5G). We then analyzed the lysosomal compartment using Lysotracker and Lamp1 staining. Confocal images revealed that the number and size of lysosomes were increased in *Tfeb^flox/flox^* astrocytes infected with the Cre virus (Figure 5H,I; Figure S8E,F, Supporting Information). ATP assays revealed that TFEB knockdown reduced astrocytic ATP release, which was reversed by ML‐SA1 (Figure 5J). Next, we constructed a recombinant plasmid with full‐length WT TFEB and transfected it into HEK293T cells. Overexpression of TFEB significantly increased *Mcoln1* mRNA levels and TRPML1 protein levels (Figure 5K,L; Figure S8G, Supporting Information). The mRNA levels of *Lamp1* and *Rab7* were also increased (Figure S8G, Supporting Information). Furthermore, the application of curcumin analog C1 (1 µM), an agonist of TFEB,^[^ ^44^ ^]^ increased ATP release from astrocytes, which was blocked by the TRPML1 antagonist ML‐SI3 (Figure 5M). Taken together, these results suggest that astrocytic TFEB in the mPFC is involved in stress vulnerability and mediates TRPML1‐dependent ATP release.

### 2.6 Overexpression of TRPML1 in the mPFC Rescues Depressive‐Like Behaviors Induced by a Reduction in Astrocytic TFEB

**Figure 6:**
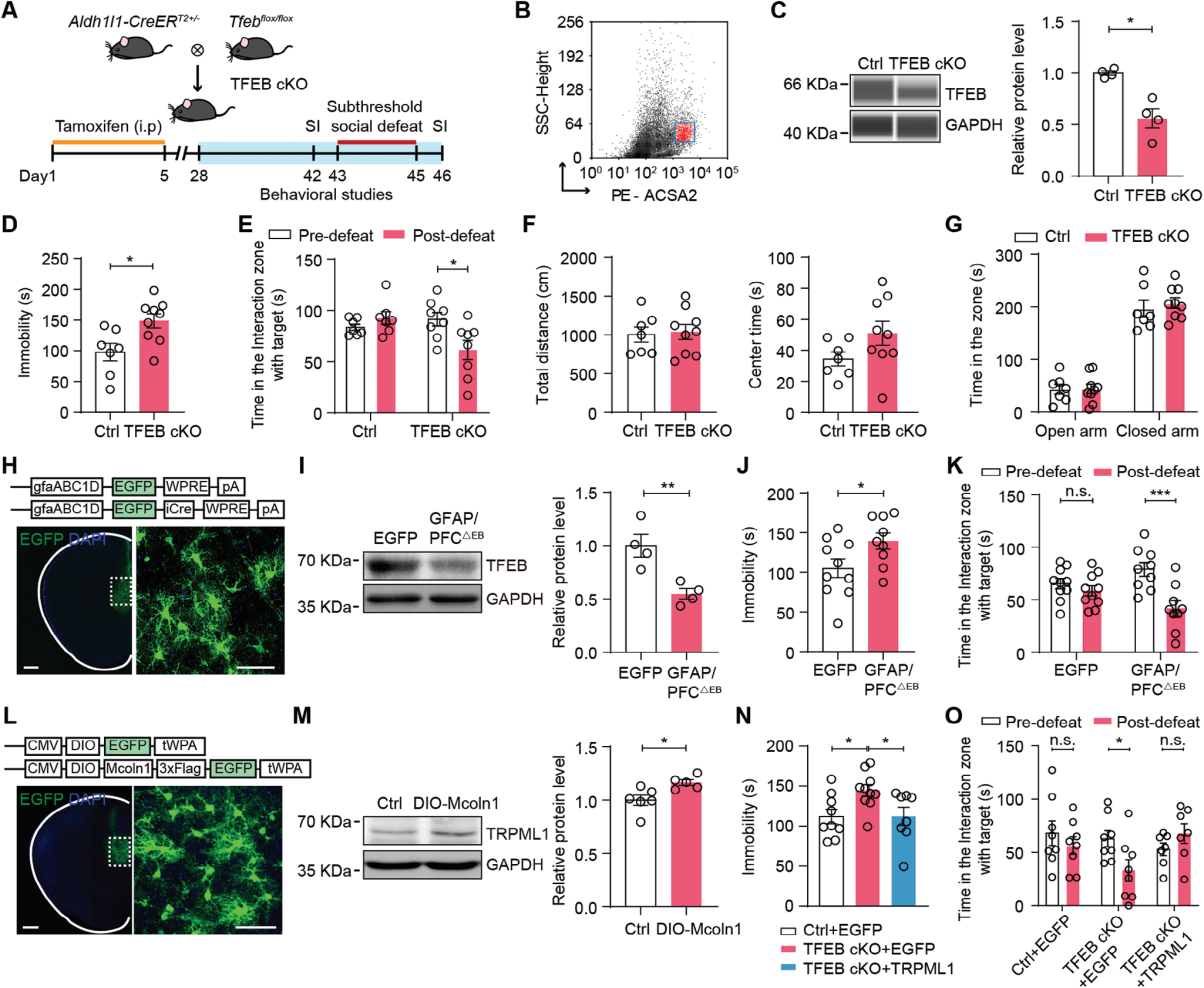
Overexpression of TRPML1 in mPFC astrocytes rescues depressive‐like behaviors in TFEB cKO mice. A) Generation of astrocyte‐specific TFEB knockout mice (TFEB cKO) and experimental timeline. B) Analysis of astrocytes isolated by FACS from the mPFC. C) Simple Western blotting analysis of astrocytic TFEB expression in the mPFC of TFEB cKO and Ctrl mice (n = 4, *p* = 0.0286). D–G) Behavioral performances of TFEB cKO and Ctrl mice in FST D) (n = 7–9, *p* = 0.0124), SI test E) (n = 7–8, *p* = 0.011), OFT F), and EPM G) (n = 7‐9). H) Representative images of AAV‐gfaABC1D‐EGFP‐iCre expression in mPFC astrocytes. Scale bars, left, 500 µm; right, 50 µm. I) Western blotting analysis of TRPML1 expression in the mPFC of GFAP/PFC^△EB^ and EGFP mice (n = 4, *p* = 0.0085). J‐K) Behavioral performances of GFAP/PFC^△EB^ and EGFP mice in FST J) and SI test K) (n = 9–10). *p* = 0.0423 J) and *p* = 0.0004 K). L) Representative images of AAV‐CMV‐Mcoln1‐3×Flag‐EGFP expression in mPFC astrocytes. Scale bars, left, 500 µm; right, 50 µm. M) Western blotting analysis of TRPML1 expression in the mPFC of TFEB cKO mice injected with AAV‐CMV‐Mcoln1‐3×Flag‐EGFP or AAV‐DIO‐EGFP virus (n = 5–6, *P* = 0.0178). N,O) Behavioral performances of TFEB cKO mice infected with AAV‐CMV‐Mcoln1‐3×Flag‐EGFP or AAV‐DIO‐EGFP virus in FST N) (n = 8–10, *p* = 0.0392 and *p* = 0.0481) and SI test O) (n = 7–8, *p* = 0.0189). Mann Whitney test C) and two‐tailed unpaired Student's t test D,F,G,I,J and M); one way ANOVA followed by Tukey's multiple comparisons test N); two‐way ANOVA followed by Sidak's multiple comparisons test E,K and O). All data are presented as the mean ± SEM. n.s., not significant; *p <0.05, **p <0.01, ***p <0.001.

To investigate the role of astrocytic TFEB in depressive‐like behaviors, we first generated astrocyte‐specific *Tfeb* deletion mice (TFEB cKO) by crossing mice expressing the floxed *Tfeb* allele with the *Aldh1l1‐CreER^T2+/−^* line (**Figure** **6**A). Astrocytes were isolated from the mPFC by FACS (Figure 6B), and Simple Western blot analysis revealed that astrocytic TFEB protein levels were decreased in the mPFC of TFEB cKO mice compared to littermate controls (Figure 6C). TFEB cKO mice presented no changes in body weight, brain size and structure, astrocyte and neuron density (Figure S9, Supporting Information). In the FST, the duration of immobility was increased in TFEB cKO mice compared to littermate controls (Figure 6D). Next, SI tests were performed before and after SSDS. There was no significant difference in social interaction time before and after SSDS in the control group (Figure 6E). In contrast, the social interaction time of TFEB cKO mice was significantly reduced after SSDS compared to before SSDS, indicating an obvious social avoidance behavior (Figure 6E). TFEB knockout did not affect general locomotion or anxiety‐related behavior (Figure 6F,G).

To determine the brain region‐specific effect of astrocytic TFEB on behavioral performances, we bilaterally injected AAV‐gfaABC1D‐EGFP‐iCre or AAV‐gfaABC1D‐EGFP virus into the mPFC of *Tfeb^flox/flox^* mice to obtain mPFC astrocyte‐specific TFEB knockout (GFAP/PFC^△EB^) or control (EGFP) mice. Three weeks after injection, confocal images revealed that astrocytes throughout the mPFC were infected with the gfaABC1D‐iCre virus (Figure 6H). Western blotting analysis confirmed a decrease in TFEB expression in the mPFC after gfaABC1D‐iCre virus injection (Figure 6I). Regarding behaviors, GFAP/PFC^△EB^ mice showed increased immobility time in the FST compared to EGFP mice (Figure 6J). After SSDS, GFAP/PFC^△EB^ mice presented decreased social interaction time in the SI test, indicating social avoidance behavior (Figure 6K). No apparent changes were observed in the EPM and OFT (Figure S10A,B, Supporting Information).

To further investigate whether overexpression of TRPML1 can rescue depressive‐like behaviors in TFEB cKO mice, we bilaterally injected AAV‐DIO‐Mcoln1‐3×Flag‐EGFP or AAV‐DIO‐EGFP virus into the mPFC of TFEB cKO and Ctrl mice and then performed behavioral tests. After three weeks, mPFC astrocytes were infected with the virus (Figure 6L), and the TRPML1 protein level was increased in the mPFC of TFEB cKO mice (Figure 6M). TFEB cKO mice infected with the control virus displayed depressive‐like phenotypes, including increased immobility time in the FST (Figure 6N) and apparent social avoidance behavior in the SI test after SSDS (Figure 6O), which were reversed by the overexpression of TRPML1 in mPFC astrocytes (Figure 6N,O). Virus infection did not affect behavioral performances in the OFT and EPM (Figure S10C–D, Supporting Information). These results suggest that overexpression of astrocytic TRPML1 in the mPFC rescues depressive‐like behaviors in TFEB cKO mice.

## 3 Discussion

In this study, we identified a stress‐sensing signaling pathway involving astrocytic lysosomes that mediates depressive‐like behaviors. We found that chronic stress altered the morphology of astrocytic lysosomes in the mPFC of susceptible mice, and the protein levels of TFEB and TRPML1 were decreased in depression models. Furthermore, the astrocytic TFEB‐TRPML1 axis modulates depressive‐like behaviors via lysosomal exocytosis‐mediated ATP release. Our work provides insight into a plausible relationship between astrocytic lysosomes and stress vulnerability to depression.

Previous studies of RNA expression in the postmortem brains of patients with MDD and bipolar disorder have shown alterations in the expression of lysosome‐related genes, including *MCOLN1* and *ATG16L2*.^[^ ^45^ ^]^ Our findings, together with previous studies, indicate that the dysfunction of astrocytic lysosomes, including lysosomal exocytosis, acidification and phagocytosis, plays an important role in depression.^[^ ^46^, ^47^ ^]^ Here, we found that chronic stress alters the morphology of astrocytic lysosomes and the expression of astrocytic TRPML1 and TFEB (Figures 1 and 5). Furthermore, astrocyte‐specific knockout of TRPML1 induced depressive‐like behaviors in mice through inhibiting lysosomal exocytosis‐mediated ATP release, establishing a direct link between astrocytic lysosomes and depression. However, neuronal TRPML1 lacks such a trait. Chronic stress enhances the release of glucocorticoid hormones into the blood, and glucocorticoid hormones in the blood could be transported across the blood‐brain barrier to the brain.^[^ ^48^, ^49^ ^]^ Our previous research has shown that astrocytes preferentially sense the stress hormones from the blood through the glucocorticoid receptor (GR), and lysosomes are associated with GR‐dependent ATP release.^[^ ^23^ ^]^ In the stress‐induced depression animal model and DXMS time‐course experiments, the TFEB‐TRPML1 axis in astrocytes is more sensitive to stress than that in neurons and microglia (Figure 1), indicating that TRPML1 in astrocytes, but not in neurons and microglia, is involved in the stress‐induced depressive‐like behaviors. Ultimately, TRPML1 deficiency in neurons did not produce depression‐related behaviors in FST and SSDS (Figure 2). Indeed, neuronal TRPML1 has been implicated in the pathogenesis of neurodegenerative diseases.^[^ ^15^, ^50^ ^]^ It might involve the cellular heterogeneity of lysosome functions and the differing contributions of lysosome functions to various brain disorders.

In the present study, TRPML1 AcKO mice presented impaired lysosomal exocytosis in astrocytes (Figure 4), which is supported by prior molecular studies.^[^ ^28^, ^29^ ^]^ TRPML1 is also involved in the modulation of autophagy processes,^[^ ^51^ ^]^ the degradation and recycling of cytoplasmic components by lysosomes. Although autophagy is closely associated with depression,^[^ ^52^ ^]^ its role in depression remains unclear. Our results showed that there were no changes in the mRNA levels of *Map1lc3a* and *Mtor*, but *Atg16l2* expression was increased in the mPFC of Res mice (Figure 1), suggesting a potential relationship between the Atg16l2‐related autophagy initiation and resilience to stress, which deserves further investigation. In addition, lysosomal acidification in astrocytes has been implicated in the resilience to chronic stress.^[^ ^46^ ^]^ Defective lysosomal acidification in microglia has been shown to increase the secretion of pro‐inflammatory cytokines,^[^ ^53^, ^54^ ^]^ which have been involved in the pathogenesis of depressive‐like phenotypes.^[^ ^5^ ^]^ The lysosomal acidification‐related components and other ion channels may contribute to the pathogenesis of depression, which deserves further investigation.

The measurements of whole‐cell and single channel currents and lysosome‐targeted genetically encoded Ca^2+^ sensors confirmed that TRPML1 channels mediate Ca^2+^ release from lysosomes in intact cells,^[^ ^55^, ^56^ ^]^ which play an essential role in most lysosomal functions including lysosomal exocytosis.^[^ ^36^ ^]^ TRPML1‐knockout cells exhibit impairment of lysosomal membrane fusion/fission, resulting in dysfunction of lysosomal exocytosis.^[^ ^25^, ^57^ ^]^ Our results revealed that astrocytic TRPML1 deficiency leads to impairment of lysosomal exocytosis‐mediated ATP release (Figure 4), suggesting that lysosomal Ca^2+^ contributes to ATP release in mPFC astrocytes from animals with depressive‐like behaviors.

Accumulated evidence has confirmed that astrocyte dysfunction drives abnormal neuronal transmission and synaptic plasticity in depression through decreased ATP release.^[^ ^22^, ^33^, ^41^, ^58^, ^59^ ^]^ ATP plays an important role in both physiological and pathological conditions by activating ionotropic P2X and metabotropic P2Y receptors on glial cells and neurons.^[^ ^60^ ^]^ ATP deficiency leads to an inhibitory/excitatory imbalance via the P2X_2_ receptor in neurons, contributing to the etiopathogenesis of depression.^[^ ^58^, ^61^ ^]^ We also detected abnormalities in neuronal calcium activity in the mPFC of TRPML1 AcKO mice under stress (Figure S7, Supporting Information), providing a potential lysosome‐related mechanism for aberrant glia‐neuronal communication in depression.

In the present study, we found that the protein levels of TFEB were decreased with no alteration of mRNA levels in the mPFC of Sus mice after CSDS (Figures 1 and 5). It might be due to the observation period, and the combined regulation of the post‐transcriptional modifications and translational processes of *Tfeb* induced by chronic stress.^[^ ^62^, ^63^, ^64^ ^]^ There is also a possibility that the decreased TFEB protein in the mPFC associated with social avoidance is not dependent on local transcriptional regulation, similar to the changes in BDNF.^[^ ^65^ ^]^ As target genes bearing the CLEAR motif regulated by TFEB, only *Mcoln1* expression, but not *Lamp1* or *Rab7* expression, was decreased in the mPFC of Sus mice (Figure 1). TFE3, another transcription factor of the MiTF/TFE family, has been reported to compensate for the loss of TFEB by regulating the target genes.^[^ ^66^, ^67^ ^]^ In addition, miRNAs play roles in the regulation of transcriptional programs during stress responses, the effects of which on TFEB‐triggered lysosomal biogenesis and autophagy have been reported.^[^ ^62^, ^63^, ^68^ ^]^ As a previous study showed that the deletion of *Tfeb* in endothelial cells does not decrease the mRNA level of *Lamp1*,^[^ ^69^ ^]^ which is similar to our results and suggests cellular heterogeneity.

Furthermore, we found that deletion of *Tfeb* in astrocytes induced a significant decrease in TRPML1 levels and enlarged lysosomal compartments (Figure 5). In contrast, some lysosomal biogenesis‐related genes were not significantly altered by *Tfeb* deficiency (Figure S8, Supporting Information). These observations suggest a dysfunction of lysosomal exocytosis, which is supported by the regulation of lysosome size by TRPML1.^[^ ^30^, ^70^ ^]^ Prior research supports this notion, showing the accumulation of Lamp1‐positive lysosomes in TFEB‐deficient cells^[^ ^71^ ^]^ and an increase in Lamp1 protein levels in TRPML1 knockout cells.^[^ ^30^ ^]^ The intensity of Lamp1 in astrocytes is increased with no changes in TFEB under depression‐related stress.^[^ ^47^ ^]^ In addition, the ability of TFE3 to compensate for the loss of TFEB, increasing the number of lysosomes has been observed in *Tfeb*‐depleted cells.^[^ ^66^, ^67^ ^]^ These results may be due to the differences in cell types and observation periods, as well as a wide range of functions regulated by TFEB, which deserves further investigation.

In conclusion, our results identify a distinct role of the lysosomal TFEB‐TRPML1 axis in depression through the regulation of lysosomal exocytosis in astrocytes. These findings reveal a plausible mechanism by which astrocytic lysosomes respond to stress and contribute to depression, and suggest the lysosome as a potential organelle target for antidepressants.

## 4 Experimental Section

### Human Subjects

Eighteen patients with MDD (eleven females and seven males) aged between 18 and 50 years were recruited through Guangdong 999 Brain Hospital, Guangzhou, Guangdong. The quantitative real‐time PCR experiment for peripheral blood of MDD patients was approved by the Ethics Committee of Guangdong 999 Brain Hospital (No. 2021‐03‐087). Twenty‐two healthy controls (fourteen females and eight males) aged between 18 and 50 years were recruited through a local community posting. All patients met the diagnostic criteria for MDD using the Structured Clinical Interview for DSM‐IV Axis I Disorders and were clinically diagnosed by two psychiatrists (You‐Lu Wen and Ting‐Ting Gu). The 24‐item Hamilton Depression Rating Scale (HAMD‐24) was used for clinical diagnosis of depression. None of these patients with first‐episode MDD had taken any psychotropic medications for longer than 3 weeks. A history of other major psychiatric disorders, head trauma, drug abuse, and insobriety were the exclusion criteria.

### Animals

All mice were housed in a temperature‐ and humidity‐controlled room with a 12 h light/dark cycle (lights on from 7:00 to 19:00) and ad libitum access to food and water. All behavioral tests were performed by observers who were blinded to the group allocations during 1:00 p.m. and 5:00 p.m. All experiments were approved by the Southern Medical University Animal Ethics Committee (No. 2016104), and conformed to the Regulations for the Administration of Affairs Concerning Experimental Animals (China).

C57BL/6J mice were obtained from the Southern Medical University Animal Center (Guangzhou, China). *Mcoln1^flox/flox^* mice were purchased from Shanghai Model Organisms Center, Inc. *Tfeb^flox/flox^* and *CamKIIa‐CreER^T2+/−^* mice were purchased from Jackson Laboratories (Stock USA). *Aldh1l1‐CreER^T2+/−^* and *Aldh1l1‐EGFP* mice were provided by Prof. Tian‐Ming Gao (Southern Medical University, China).

For the excision of the flox sites by Cre recombination, 8‐week‐old male mice were injected intraperitoneally with tamoxifen (TAM) (Sigma–Aldrich, USA) once a day (100 mg kg^−1^ of body weight) for 5 consecutive days. TAM was dissolved in corn oil (Sigma, USA) to a final concentration of 10 mg ml^−1^. Behavioral tests were performed four weeks after TAM injection.

### Transmission Electron Microscope

Mice were rapidly anesthetized with isoflurane. The mPFC tissues (1 mm^3^ in size) were immediately dissected from the mouse brain within 1 min and fixed in a 2.5% glutaraldehyde solution at 4 °C overnight. The tissues were then rinsed in PBS and post‐fixed in 1% osmium tetroxide. After a series of acetone dehydration and Epox 812 infiltration, the tissues were dehydrated in increasing concentrations of alcohol and then embedded in durcupan resin and cured at 65 °C for 24 h. Next, ultrathin sections (80 nm thick) were cut using an ultramicrotome (Leica, UC7). Finally, images were captured using a transmission electron microscope (Hitachi H‐7500, Japan) operated at 60 kV, and analyzed using ImageJ software.

### Immunofluorescence

Mice were deeply anesthetized with isoflurane and perfused transcardially with saline followed by 4% paraformaldehyde (PFA) in 0.1 M PBS (pH 7.4). Brains were removed and post‐fixed in 4% PFA at 4 °C overnight. Brains were then transferred to 30% sucrose solution in 0.1 M PBS and dehydrated to isotonicity. After three days of dehydration, coronal sections (40 µm) were cut on a freezing microtome (Leica, CM1950, Germany) at −20 °C. For cell staining, primary astrocytes cultured on coverslips were fixed in 4% paraformaldehyde for 15 min. The samples were washed three times with 0.1 M PBS before incubation with blocking buffer containing 5% normal goat serum in 0.5% Triton X‐100/PBS (permeabilization) for 1.5 h at room temperature. The surface expression of Lamp1 was detected in non‐permeabilized astrocytes. Cells or slices were incubated overnight at 4 °C with primary antibodies: mouse‐anti Lamp1 (1:100, Abcam), rabbit‐anti TRPML1 (1:500, Abcam), rabbit‐anti TFEB (1:500, Bethyl), mouse‐anti NeuN (1:500, Cell Signaling), mouse‐anti S100β (1:500, Abcam), guinea pig‐anti S100β (1:500, Synaptic Systems), mouse‐anti GFAP (1:500, Cell Signaling), goat‐anti Iba1 (1:500, Novus Biologicals). The samples were then washed three times with PBS, and incubated with secondary antibodies for 2 h at room temperature. Finally, the samples were counterstained with 4, 6‐diamidino‐2‐phenylindole (DAPI) (Vector Laboratories, CA, USA) for nuclei staining. Images were acquired and visualized with a Nikon A1R confocal microscope (Nikon Instruments Inc., Japan).

To quantify the fluorescence intensity of Lamp1 in mPFC astrocytes, 20‐µm coronal slices were stained with Lamp1 (1:100, Abcam) for 24 h, followed by Alexa Fluor 594‐conjugated secondary antibody staining. Next, the slices were stained with DAPI. Imaging was performed on an N‐SIM microscope (Nikon Instruments Inc., Japan) using a 100 × oil objective. Z stacking was performed with 0.1‐µm steps in the z direction, and images were analyzed using Imaris 8.1 software.

### Quantitative Real‐Time PCR

The isolated tissue samples and white blood cell samples were immediately stored in TRIzol (Takara, Japan), and RNA was extracted according to the manufacturer's instructions. Then, cDNAs were synthesized using the PrimeScriptTM RT Reagent Kit (Takara, Japan) according to the protocol supplied by the manufacturer. The Quantitative real‐time PCR (qRT‐PCRs) were performed using a 7500 real‐time PCR system (ABI, USA). Each reaction was performed in duplicate. The housekeeping gene *Gapdh* was used as an internal control for normalization with the _∆∆_Ct method. The 2−_∆∆_Ct method was used to calculate the relative expression levels of the genes. Primer sequences used for qRT‐PCRs were listed in Table S1 (Supporting Information).

### Fluorescent‐Activated Cell Sorting (FACS)

Mice were anesthetized with isoflurane and perfused with ice‐cold artificial cerebrospinal fluid (ACSF) solution (containing (in mM) 125 NaCl, 2.5 KCl, 2 CaCl_2_, 2 MgSO_4_, 1.25 NaH_2_ PO_4_, 26 NaHCO_3_, and 10 D‐glucose). Brains were dissected and cut in ice‐cold oxygenized ACSF using a Vibroslice (Leica, Germany). One sample consisted of mPFC brain slices isolated from two mice. The slices separated from the brain were then coarsely chopped, and incubated in 5 ml EBSS solution containing 2 mg ml^−1^ papain (Sigma, USA), 1 mg ml^−1^ L‐cysteine (Sigma, USA), 0.5 mg ml^−1^ EDTA (Sigma, USA), and 100 µg ml^−1^ DNase I (Sigma, USA) for 30 min at 37 °C. During the cell dissociation, brain tissues were blown with an 800‐µm pipette ≈15 times every 15 min. Then, the cell pellet was resuspended in 5 ml of a 26% Percoll solution and centrifuged at 900 g (4 °C) for 15 min. A 40‐µm mesh was used to eliminate cell clumps. The cells were incubated in FACS buffer containing FcR blocking reagent (Miltenyi, USA) and an anti‐ACSA‐2‐PE monoclonal antibody (Miltenyi, USA) for 30 min on ice in the absence of light. Finally, the cells were analyzed and collected using a MoFlo XDP flow cytometer (Beckman Coulter, USA).

### Magnetic‐Activated Cell Sorting (MACS)

Adult mice were anesthetized with isoflurane and perfused through the heart with ice‐cold saline. The mPFC was then immediately removed and washed with prechilled phosphate‐buffered saline (PBS). The mPFC tissues were mechanically and enzymatically dissected using the Adult Mouse Brain Dissociation Kit (Miltenyi Biotec) in the single cell suspensions instrument. Myelin‐free single cell suspensions were obtained after removal of myelin according to the cell debris removal buffer in the kit, and the whole cell pellet was resuspended with PBS containing 0.5% BSA, followed by isolation of the desired cell type. Briefly, astrocytes were isolated using anti‐ACSA‐2 MicroBeads (Miltenyi Biotec) and adult neurons were isolated using the Adult Neuron Isolation kit (Miltenyi Biotec), and then both were separated by magnetic columns. The isolated cells were placed in a −80 °C refrigerator immediately after centrifugation.

### Cell Culture, Transfection and Drug Administration

For primary astrocyte culture, the tissues isolated from mouse cerebral cortex (P1‐3) were washed with ice‐cold PBS and dissociated using a pair of sterile operating scissors in a 50 ml Falcon tube containing 0.5 ml PBS. The tissues were then incubated with 0.25% trypsin (Thermo Fisher Scientific, USA) containing 0.5 mM EDTA for 10 min at a temperature of 37 °C. To inhibit the trypsin activity, the culture medium (DMEM/F‐12 with 10% fetal bovine serum (FBS)) (CORNING, USA) was added, and then the cell suspension was centrifuged at 900 × g for 5 min. After being resuspended in 10 ml of culture medium, the cells were transferred to a culture flask at a density of 5×10^6^ cells per 5 ml. They were then incubated in a humidified incubator with 5% CO_2_ at 37 °C.

For primary neuron culture, fetal mice were dissected from timed‐pregnant C57BL/6J females at E16.5. The tissues were separated from the cerebral cortex of fetal mice, and dissociated by enzymatic digestion. After inhibiting trypsin activity, the cell suspension was centrifuged at 900 × g for 5 min. Isolated primary neurons were cultured on poly‐D‐lysine‐coated dishes using Neurobasal medium supplemented with B27 (GIBCO, USA) and incubated in a humidified CO_2_ incubator with 5% CO_2_ at 37 °C.

Flag‐EGFP‐tagged full‐length TFEB plasmids were constructed (OBio Bioscience, China). H293T cells (ACTT, USA) were grown to 70% confluence and transfected using TransIT‐X2 (Mirus Bio, USA) according to the manufacturer's protocol. Cells were transfected at 37 °C for 24 h.

For Western blotting, primary astrocytes were cultured for 8 days while neurons were cultured for 14 days before drug administration. BV2 cells were cultured and proliferated to 70% of cell density. Cells were treated with DXMS (1 µM) for 0, 4, 12, 24, and 48 h, respectively, and then collected for Western blotting analysis.

### Western Blotting Analysis

Proteins were extracted from brain tissues or cultured cells, which were homogenized in lysis buffer (RIPA) (Thermo Fisher Scientific, USA) containing protease inhibitor (PMSF) (Thermo Fisher Scientific, USA) and centrifuged at 12 000 rpm at 4 °C for 30 min to remove insoluble material. Protein concentration was then measured and quantified using the bicinchoninic acid (BCA) protein assay kit (Thermo Fisher Scientific, USA). Samples were loaded and separated on 10% sodium dodecyl sulfate‐polyacrylamide gel electrophoresis (SDS‐PAGE) gels and then electrotransferred on PVDF membranes (Millipore, Germany) in ice‐cold buffer (25 mM Tris‐HCl, 192 mM glycine, and 20% methanol) for 2 h. Membranes were blocked with 5% nonfat milk for 1 h at room temperature and then incubated overnight at 4 °C with the following primary antibodies: rabbit anti‐TRPML1 (1:1000, Abcam), rabbit anti‐TFEB (1:1000, Bethyl), rabbit anti‐Phospho TFEB (S211) (1:1000, Cell Signaling), rabbit anti‐Lamp1 (1:1000, Abcam), rabbit anti‐Rab7 (1:1000, Abcam), mouse anti‐GAPDH (1:5000, Proteintech), goat anti‐LaminB (1:1000, Santa Cruz Biotechnology). Membranes were washed three times with TBS buffer containing 0.1% Tween‐20 (TBST) and incubated with HRP‐conjugated secondary antibodies for 1 h at room temperature. After three washes with TBST, the membranes were imaged using a BIO‐RAD Gel Doc XR imaging system (BIO‐RAD, Germany) and quantitatively analyzed using Image Lab software. For analysis, protein levels were normalized to GAPDH on the same gel.

### Simple Western Blot Analysis

Astrocytes isolated from the mPFC by FACS were lysed in RIPA buffer containing 1% PMSF. Samples were mixed with sample buffer to a final concentration of 1 µg µl^−1^. The Simple Western system (ProteinSimple, USA) was then used for protein detection. Target proteins were identified with the following primary antibodies: rabbit anti‐TRPML1 (1:100, Abcam); rabbit anti‐TFEB (1:100, Bethyl); mouse anti‐GAPDH (1:100, Proteintech). Horseradish peroxidase (HRP)‐conjugated secondary antibodies and chemiluminescent substrates were used for subsequent immunodetection.

### Extracellular ATP, AP and β‐Gal Measurements

To reduce ATP hydrolysis, the ectonucleotidase inhibitor 6‐N, *N*‐diethyl‐β‐γ‐dibromomethylene‐d‐adenosine‐5‐triphosphate FPL 67156 (ARL 67156 trisodium salt) (Sigma–Aldrich, USA) was added to the extracellular medium or ACSF throughout the experiment. The extracellular medium or ACSF was then transferred to a centrifuge tube and cell debris was removed by centrifugation. ATP levels were measured using a bioluminescent ATP assay kit (Promega, USA). Acid phosphatase (AP) and β‐galactosidase (β‐Gal) activities were measured using the AP colorimetric assay kit (Abcam, USA) and β‐galactosidase assay kit (Abcam, USA), respectively. Luminescence was measured using a luminometer (PerkinElmer, USA) according to the manufacturer's instructions. For quantification, ATP, AP and β‐Gal amounts were normalized to the amount of total protein in each sample using the BCA protein assay kit (Thermo Fisher Scientific, USA).

### Time‐Lapse Imaging

Virus‐transfected astrocytes were loaded with FM2‐10 (50 µM) in DMEM/F‐12 containing 10% FBS for 2 h at 37 °C. After washing for in PBS 20 min, astrocytes were transferred to the confocal dish (CORNING, USA) for imaging under a confocal microscope with a 40 × water‐immersion objective. Before and after glutamate treatment, the time courses of the changes in fluorescence of FM2‐10 with λ _emission_ > 560 nm were obtained at an image interval of 1 second. The decrease in the fluorescence intensity of the FM caused by photobleaching was less than 5% over a period of 10 min. Data analysis was performed with NIS Elements (Nikon, Japan).

### Chromatin Immunoprecipitation Assay

Chromatin immunoprecipitation assay (ChIP) was performed using a SimpleChIP Enzymatic Chromatin IP Kit (Cell signaling, USA) according to the manufacturer's protocol. Chromatin/DNA protein complexes of astrocytes were obtained by cross‐linking with 1% formaldehyde by incubation at a room temperature for 20 min. Cell extracts were then sonicated to generate chromatin fragments of 150–900 bp in length, and incubated with rabbit anti‐TFEB antibody (1:50, Cell Signaling) or IgG overnight at 4 °C with constant rotation. After incubation, 30 µL of Protein G bead slurry was added to the lysates and incubated for 2 h at 4 °C with rotation. The resins were washed 3 times with ChIP buffer. Chromatin fragments were eluted from antibody/Protein G beads with a ChIP elution buffer, and cross‐linking was reversed with 5 M NaCl and 2 µl of 20 mg ml^−1^ Proteinase K. Finally, PCR was performed using primers designed to cover the putative TFEB/CLEAR sequence in the mouse *Mcoln1* promoter.

### Stereotaxic Surgery

Mice were anesthetized with isoflurane and then fixed in a stereotactic frame (RWD Life Science, China) before surgery. Coordinates were measured from the bregma according to the mouse atlas. A volume of 300 nl of virus was injected into the bilateral mPFC (anterior‐posterior (AP): +1.75 mm; medial‐lateral (ML): ± 0.3 mm; dorsal‐ventral (DV): – 2.5 mm) at a rate of 100 nl min^−1^. The needle was left in place for 6 min after each injection to allow the virus to diffuse. The needle was then slowly withdrawn, and the scalp was sutured.

For specific knockdown of astrocytic TRPML1 or TFEB, a virus containing Cre under a gfaABC1D promoter (AAV2/9‐gfaABC1D‐EGFP‐iCre‐WPRE‐pA) or a control virus (AAV2/9‐gfaABC1D‐EGFP‐WPRE‐pA), purchased from Taitool Bioscience (China), was bilaterally injected into the mPFC of *Mcoln1^flox/flox^* or *Tfeb^flox/flox^* mice.

To overexpress TRPML1 in astrocytes, an AAV2/9‐CMV‐DIO‐Mcoln1‐3×Flag‐P2A‐EGFP‐tWPA virus or an AAV2/9‐CMV‐DIO‐EGFP‐tWPA virus purchased from OBiO Bioscience was bilaterally injected into the mPFC of *Aldh1l1‐CreER^T2+/−^; Mcoln1^flox/ flox^* or *Aldh1l1‐CreER^T2+/−^; Tfeb^flox/ flox^* mice.

For fiber photometry, AAV‐GfaABC1D‐ATP1.0 or AAV‐CamKIIa‐Gcamp6s virus was unilaterally injected into the mPFC of *Aldh1l1‐CreER^T2+/−^; Mcoln1^flox/ flox^* mice. A ceramic ferrule with an optical fiber (200 µm diameter, 0.37 numerical aperture (Inper, China)) was then implanted over the infected cells of the mPFC (AP: + 1.75 mm; ML: ± 0.3 mm; DV: – 2.5 mm) after virus injection. Three weeks after fiber implantation, fluorescence signals were recorded.

### Fiber Photometry

After 2 weeks for viral expression, fiber photometry was used to record ATP or calcium signals during the forced interaction test using a commercialized system. The laser beam emanated from the laser tube (488 nm) is reflected by a dichroic mirror focused by a 10× (NA = 0.3) lens and coupled to an optical commutator. The fluorescence was bandpass filtered (Thorlabs, USA) and collected with a photomultiplier tube (Hamamatsu, Japan). An amplifier (Hamamatsu, Japan) was used to convert the current output of the photomultiplier into a voltage signal, which was further filtered by a low‐pass filter. The laser power at the fiber tip was adjusted to 30 µW to minimize photobleaching. The optical fiber was connected to the fiber photometry system using an optical fiber sleeve. The videos were recorded and analyzed to determine the time of interaction of CD1 mice with TRPML1 AcKO or Ctrl mice. Signal processing was performed using MATLAB (MathWorks, USA). The z‐score of population of astrocytes or neurons was calculated using the following formula: z‐score = (F_Signal –_ F_Basal_) / STD (F_Basal_).

### Chronic Social Defeat Stress

Chronic social defeat stress (CSDS) was performed according to previous protocols.^[^ ^21^ ^]^ Prior to the start of defeat, CD1 mice (aggressor) were screened for aggressive behavior and then placed on one side of a cage with a perforated translucent plastic divider (0.6 cm × 46 cm × 15 cm). For 10 consecutive days, the adult male C57BL/6J mouse or Aldh1l1‐EGFP mouse was exposed to a different CD1 mouse for 10 min each day. After 10 min of physical interaction, the CD1 mouse and the test mouse were separated by a perforated translucent plastic divider, allowing continuous chronic stress to persist in the form of a threat for the next 24 h. Control mice were housed in equivalent cages with members of the same strain and rotated daily to an unfamiliar mouse, without being exposure to CD1 mice. The social interaction (SI) test was performed to test avoidance behavior after the last bout of physical interaction.

### Subthreshold Social Defeat Stress

This procedure was identical to the normal chronic social defeat stress procedure, except that the procedure lasted for three consecutive days.

### Social Interaction Test (SI)

Social avoidance behavior was measured using a two‐stage social interaction test. Mice were placed in an open field arena (42 cm × 42 cm × 42 cm) containing an empty plexiglass cage (9.5 cm × 9.5 cm × 8 cm) on one side. Their movements were tracked and recorded by a video tracking system (Ethovision XT, Noldus Information Technology, Netherlands) for 2.5 min in the absence of the caged aggressor (“no target”). Their movements were then tracked for 2.5 min in the presence of the aggressor (“target”). The arena was cleaned with 75% ethanol after each trial to remove olfactory cues. All behavioral tests were conducted in the darkness. The interaction ratio was calculated as [(interaction time, “target”) / (interaction time, “no target”)]. Mice with an interaction ratio <1 were considered susceptible, and those with an interaction ratio ≥1 were considered resilient. Mice that with open wounds greater than 1 cm as a result of repeated defeats were excluded.^[^ ^21^ ^]^

### Forced Interaction Test

Mice were placed in an 8 cm × 15 cm Plexiglas cylinder which was located in the center of a cage. A lid was placed over the cylinder to prevent the aggressor from climbing in. The fluorescence signal was recorded for 5 min. The CD1 aggressor mouse was then placed in the cage outside of the cylinder. Fluorescence signals were recorded for a further 5 min. All mice were subjected to forced interaction test (FIT) both before and after exposure to SSDS.

### Forced Swimming Test

After 30 min of habituation, mice were individually placed into a clear glass cylinder (height 45 cm, diameter 19 cm) filled with water to a height of 23 cm (23 to 25 °C). The mice swam for 6 min under normal light conditions. The immobility time during the last 4 min of the test was measured with a tracking system (Ethovision XT, Noldus Information Technology, Netherlands). Increasing immobility in mice was used to measure behavioral despair, while decreasing immobility was considered to have an anti‐depressant effect. Under drug treatment conditions, the forced swimming test (FST) was performed 30 min after the injection.

### Sucrose Preference Test

Mice were individually housed and habituated with two 60 ml tubes with stoppers filled with water for 2 days, followed by two bottles of 1% sucrose for 2 days. After habituation, mice were exposed to one bottle of water and one bottle of 1% sucrose solution for 24 h. Bottle positions were switched each day. The sucrose preference was calculated for each mouse using the formula 100 × (total consumption of sucrose/total consumption of both water and sucrose). Data from one TRPML1 AcKO mouse were excluded due to a place preference.

### Elevated Plus Maze (EPM)

The apparatus consists of a central platform (5 cm × 5 cm) and two open arms (30 cm × 5 cm× 0.5 cm) and two closed arms (30 cm × 5 cm × 15 cm) connected by the platform. The apparatus was positioned 50 cm above the ground. After 30 min of habituation, mice were placed in the middle platform of the two open arms and allowed to explore freely for 5 min. The time spent in the open arms and closed arms was tracked and recorded by Ethovision XT.

### Open Field Test (OFT)

The open field apparatus consists of a rectangular chamber (40 cm × 40 cm × 30 cm) made of gray polyvinyl chloride. After the 30 min habituation period, mice were placed in the center of the apparatus and allowed to explore freely for 5 min. Cumulative time spent in the center region and total distance were recorded using an automated SuperFlex Open Field system (Omnitech, USA) and analyzed using Fusion 6.47 software (USA).

### Rotarod Test

A rotarod training system (Med‐associates, USA) was used for motor learning. Mice were placed on the stationary rod for 2 min for habituation. During the training period, mice were placed on the rod at a fixed speed (8 rpm), and the speed was then uniformly accelerated to 40 rpm/min after the mice remained reliably on the rod. Three training sessions were performed over 2 days. To assess learning, the procedure (4 to 40 rpm) was repeated for 3 consecutive trials. The latency to fall (in seconds) was recorded.

### Pole Climbing Test

The pole test was performed as previously described.^[^ ^72^ ^]^ Mice were placed top of a vertical pole (diameter: 1 cm, height: 55 cm) with head‐down and allowed to descend. This training was repeated 4–6 times. In the next regular turning and descending procedure, mice were trained 3–4 times by being placed on top of the pole with their head up. Then, the mice were performed eight trials and the maximum duration of each session is 30 s. The time to orient downward and the total time to turn and descend the pole were recorded. The average of the eight trials was used as the final score.

### Statistical Analysis

All statistical analyses were performed using SPSS 22.0 and GraphPad Prism 9.3.1 (GraphPad Software, USA). All data were presented as the mean ± SEM. Data were confirmed to be normally distributed using the Shapiro‐Wilk test. Two‐tailed unpaired Student's t‐tests or Mann‐Whitney U tests were used to evaluate statistical significance of differences between the two groups. The following statistical tests were used for the comparisons between more than two groups: One way ANOVA followed by Dunnett's or Tukey's multiple comparisons, Kruskal‐Wallis test followed by Dunn's multiple comparison, two‐way ANOVA followed by Sidak's multiple comparisons. Differences were considered to be significant if *p* <0.05. Significance levels were indicated as follows: *p <0.05, **p <0.01, ***p <0.001 and ****p <0.0001.

## Conflict of Interest

The authors declare no conflict of interest.

## Author Contributions

J.‐W.M. and P.‐L.K. contributed equally to this work. X.C. conceived and supervised the project. X.C., J.‐W.M. and P.‐L.K. designed the research and wrote the paper. J.‐W.M., J.F., R.M., F.G., and L.D. performed behavioral experiments and stereotactic injection with the help of C.‐L.L. and J.R. J.‐W.M., P.‐L.K. and Q.‐L.Z. performed immunostaining, Western blotting and qRT‐PCR analysis. J.‐W.M. and J.F. performed FACS and Simple Western blot analysis. P.‐L.K., L.‐Y.C. performed cell culture with the help of S.‐J.L. and T.G. Y.‐L.W. and T.‐T.G. collected blood from MDD patients. J.‐W.M. and Q.‐W.W. analyzed the data. T.‐M.G. provided suggestions for this research. All authors reviewed the manuscript.

## Supporting information



Supporting Information

Supporting Data

## Data Availability

The data that support the findings of this study are available from the corresponding author upon reasonable request.
