# Supplementary material for: Lysosomal TFEB‐TRPML1 Axis in Astrocytes Modulates Depressive‐like Behaviors
Source: Adv Sci (Weinh). 2024 Sep 12;11(41):2403389. doi: 10.1002/advs.202403389 (PMC11538709; doi:10.1002/advs.202403389)
Supplement: Supplementary file 1 — Supporting Information [file ADVS-11-2403389-s001.docx]

Supporting Information

**Lysosomal TFEB-TRPML1 axis in astrocytes modulates depressive-like behaviors**

*Jia-Wen Mo, Peng-Li Kong, Li Ding, Jun Fan, Jing Ren, Cheng-Lin Lu, Fang Guo, Liang-Yu Chen, Ran Mo, Qiu-Ling Zhong, You-Lu Wen, Ting-Ting Gu, Qian-Wen Wang, Shu-Ji Li, Ting Guo, Tian-Ming Gao, Xiong Cao**


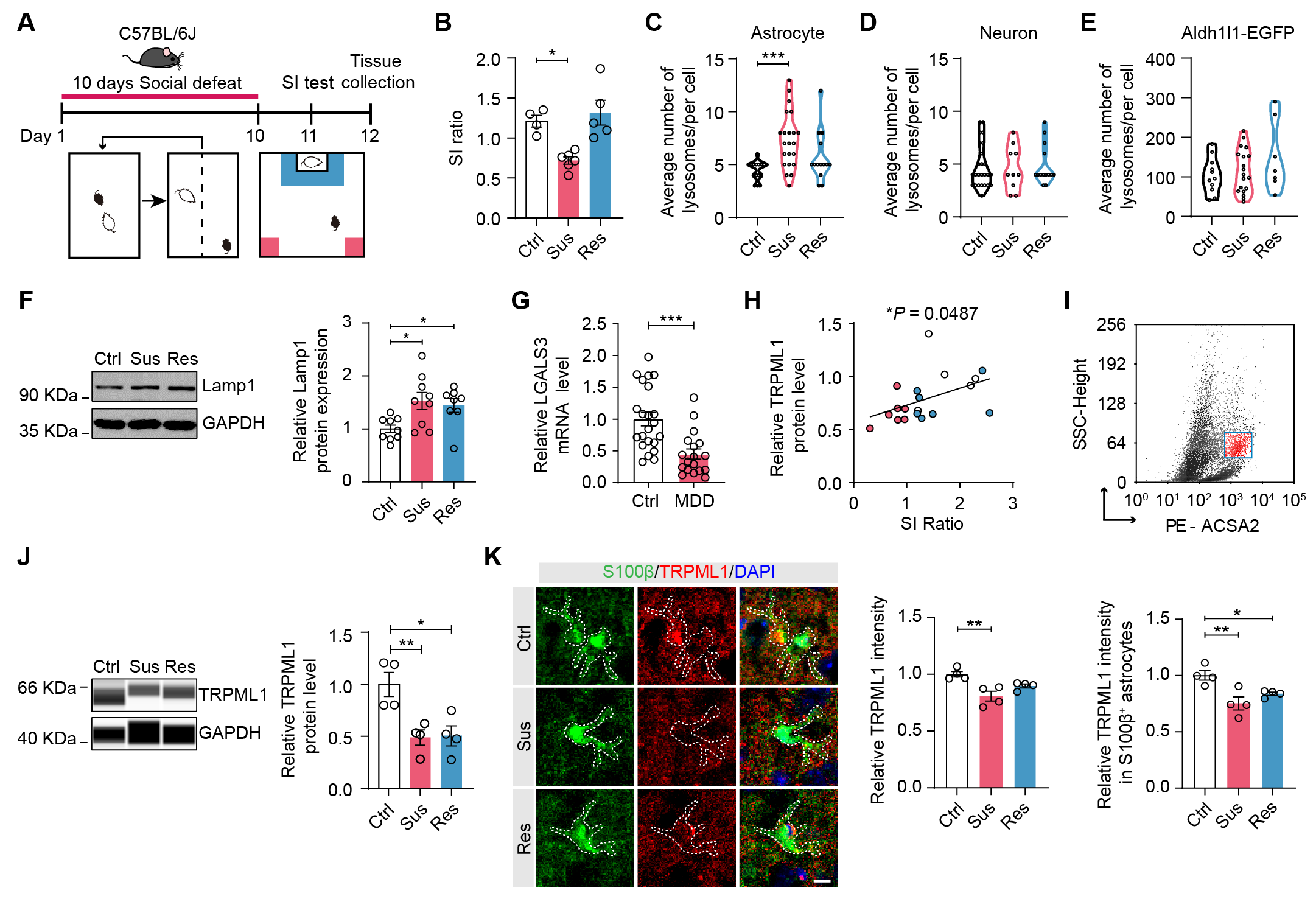
Figure S1. Chronic stress alters lysosomal morphology and decreases TRPML1 levels in mPFC astrocytes. (A) Chronic social defeat stress (CSDS) and social interaction (SI) test protocols. (B) SI ratio of Sus, Res, and Ctrl mice after the CSDS paradigm (n = 4-6, *P* = 0.0109). (C-D) Quantification of lysosomal number in the mPFC of Ctrl, Sus and Res mice (astrocyte: n = 16 cells from 4 Ctrl mice, n = 22 cells from 6 Sus mice, n = 14 cells from 5 Res mice, *P* = 0.0003; neuron: n = 25 cells from 4 Ctrl mice, n = 10 cells from 6 Sus mice, n = 14 cells from 5 Res mice). (E) Quantification of lysosomal number in mPFC astrocytes (Aldh1l1-EGFP) from Ctrl, Sus and Res mice (n = 11 cells from 3 Ctrl mice, n = 19 cells from 3 Sus mice, n = 7 cells from 3 Res mice). (F) Protein levels of Lamp1 in the mPFC of Ctrl, Sus and Res mice (n = 8-9, *P* = 0.0124 and *P* = 0.042). (G) *LGALS3* mRNA levels in the peripheral blood of MDD patients and healthy controls (n = 18-22, each sample was analyzed in duplicate, *P* = 0.0003). (H) Correlation between the TRPML1 protein level and the SI ratio. *P* = 0.0487. (I) Representative analysis of astrocytes isolated by FACS from the mPFC of C57BL/6J mice following the CSDS paradigm. (J) Simple Western blot analysis of TRPML1 protein levels in mPFC astrocytes of C57BL/6J mice after the CSDS paradigm (n = 4, *P* = 0.0086 and *P* = 0.0103). (K) Representative images and quantification of the coexpression of TRPML1 (red) and S100β (green) in the mPFC of C57BL/6J mice after the CSDS paradigm (n = 4 mice). *P* = 0.0029 (left), *P* = 0.0046 and *P* = 0.0422 (right). Scale bars, 10 µm. One way ANOVA followed by Dunnett’s post-hot test (B, E, F, J and K); Kruskal-Wallis test followed by Dunn's multiple comparisons test (C and D); two-tailed unpaired Student’s t test (G) and correlations evaluated with the Pearson correlation coefficient (H). All data are presented as the mean ± SEM. **P* < 0.05, ***P* < 0.01, ****P* < 0.001.

**
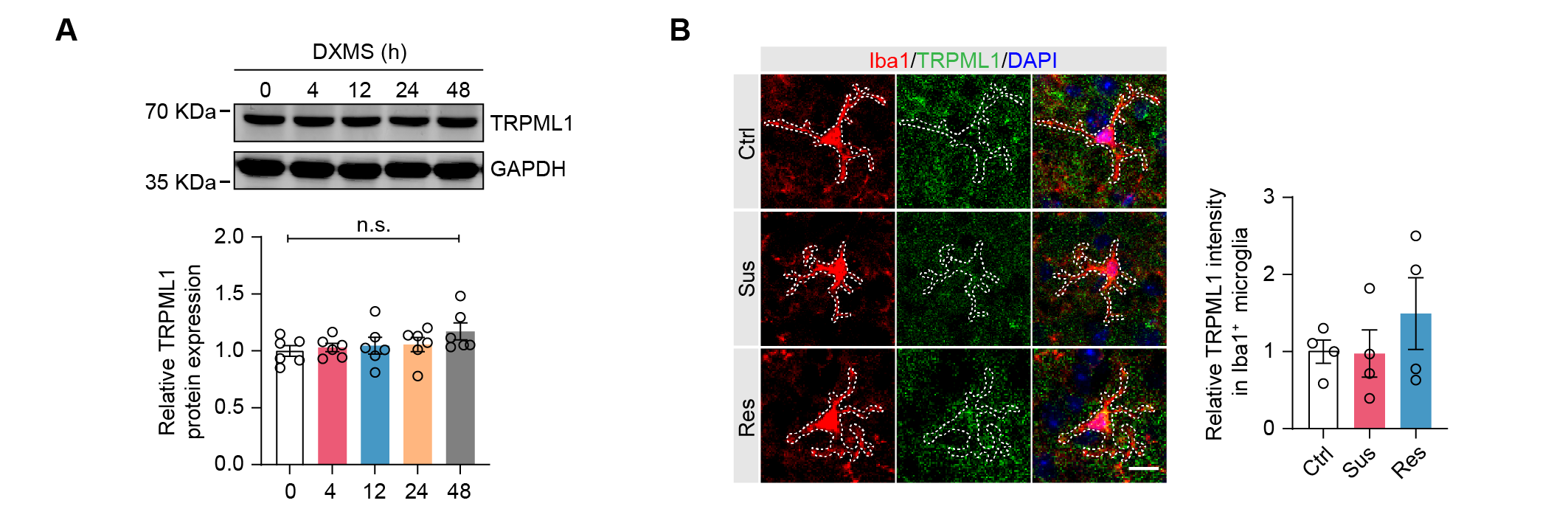
****Figure S2.** **Chronic stress does not affect the expression of TRPML1 in microglia.** (A) Western blotting analysis of TRPML1 protein levels in cultured microglial BV2 cells treated with DXMS (1 μM) for 0, 4, 12, 24, or 48 hours (n = 6). (B) Representative images and quantification of the coexpression of TRPML1 (green) and Iba1 (red) in the mPFC of C57BL/6J mice following the CSDS paradigm (n = 4 mice). Scale bars, 10 µm. One way ANOVA followed by Dunnett’s post-hot test (A and B). All data are presented as the mean ± SEM. n.s., not significant.


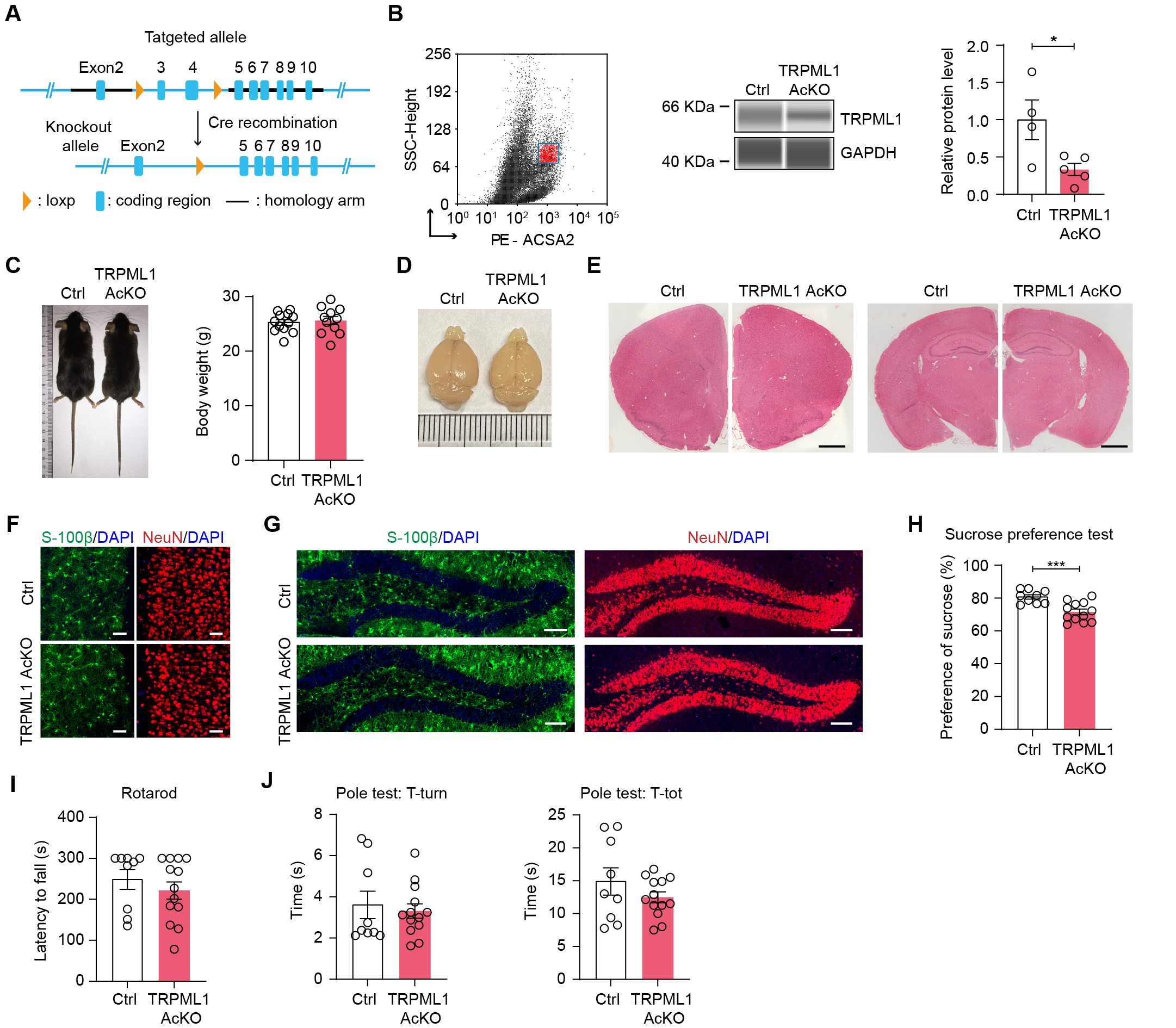


Figure S3. Generation of *Aldh1l1-CreER^T2+/-^; Mcoln1^flox/flox^* mice. (A) Schematic of the *Mcoln1^flox/flox^* allele. (B) Analysis of astrocytes isolated by FACS from the mPFC (left) and Simple Western blotting analysis of TRPML1 expression in mPFC astrocytes of TRPML1 AcKO and Ctrl mice (n = 4-5, *P* = 0.0324). (C) Body weight of TRPML1 AcKO and littermate control mice after TAM injection (n = 11-12). (D) Representative image of the brain size of TRPML1 AcKO and littermate control mice after TAM injection. (E) Hematoxylin and eosin (H&E) staining of the mPFC and hippocampus of TRPML1 AcKO and Ctrl mice. Scale bars, 1000 µm. (F-G) Immunofluorescence staining of S100β (green) and NeuN (red) in the mPFC (F) and hippocampus (G) of TRPML1 AcKO and Ctrl mice. Astrocytes were stained with S100β and DAPI; neurons were stained with NeuN and DAPI. Scale bars = 50 μm (F); 100 μm (G). (H) Behavioral performances of TRPML1 AcKO and Ctrl mice in the sucrose preference test (n = 9-12, *P* = 0.0008). (I-J) Behavioral performances of TRPML1 AcKO and Ctrl mice in the rotarod and pole climbing tests (n = 9-13). Two-tailed unpaired Student’s t test (B, C, H, I and J). All data are presented as the mean ± SEM. **P* < 0.05, ****P* < 0.001.


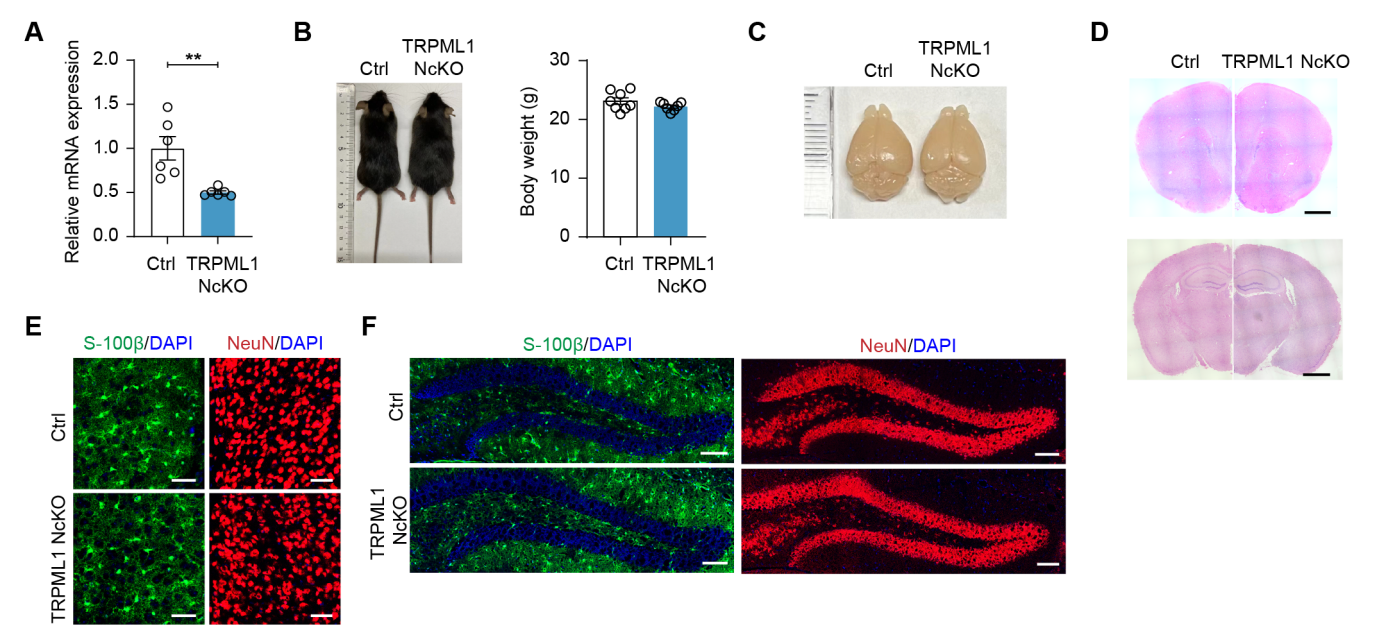


Figure S4. Generation of *CamKIIa-CreER^T2+/-^; Mcoln1^flox/flox^* mice. (A) *Mcoln1* mRNA levels in mPFC neurons collected by MACS from TRPML1 NcKO and Ctrl mice (n = 6, *P* = 0.0022). (B) Body weight of TRPML1 NcKO and littermate control mice after TAM injection (n = 7-8). (C) Representative image of the brain size of TRPML1 NcKO and littermate control mice after TAM injection. (D) H&E staining of the mPFC and hippocampus of TRPML1 NcKO and Ctrl mice. Scale bars, 1000 µm. (E-F) Immunofluorescence staining of S100β (green) and NeuN (red) in the mPFC (E) and hippocampus (F) of TRPML1 NcKO and control mice. Astrocytes were stained with S100β and DAPI; neurons were stained with NeuN and DAPI. Scale bars = 50 μm (E); 100 μm (F). Mann Whitney test (A); two-tailed unpaired Student’s t test (B). All data are presented as the mean ± SEM. ***P* < 0.01.


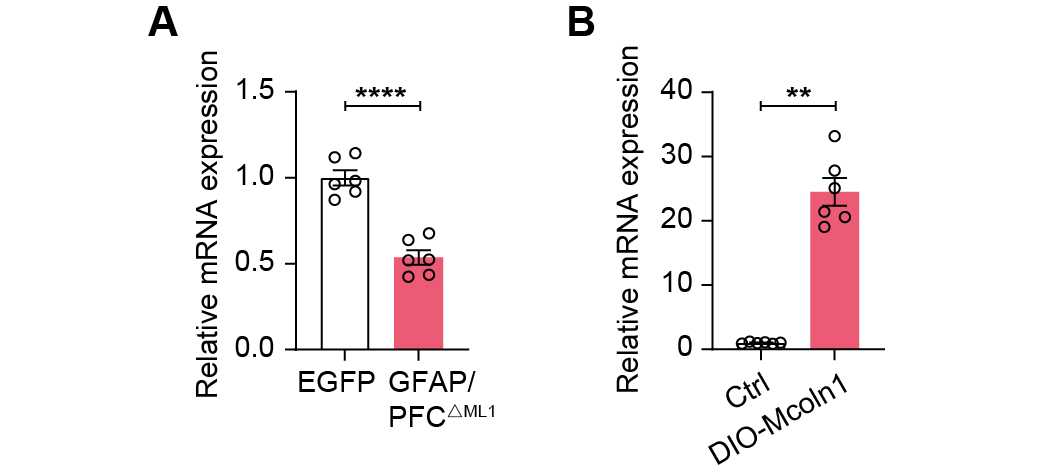


**Figure S5**. ***Mcoln1* mRNA levels in mPFC astrocytes collected by MACS.** (A) *Mcoln1* mRNA levels in mPFC astrocytes isolated from GFAP/PFC^△ML1^ and EGFP mice (n = 6, *P* < 0.0001). (B) *Mcoln1* mRNA levels in mPFC astrocytes isolated from TRPML1 AcKO mice infected with AAV-DIO-Mcoln1-3×Flag-EGFP or AAV-DIO-EGFP virus (n = 6, *P* = 0.0022). Two-tailed unpaired Student’s t test (A); Mann Whitney test (B). All data are presented as the mean ± SEM. ***P* < 0.01, *****P* < 0.0001.

**
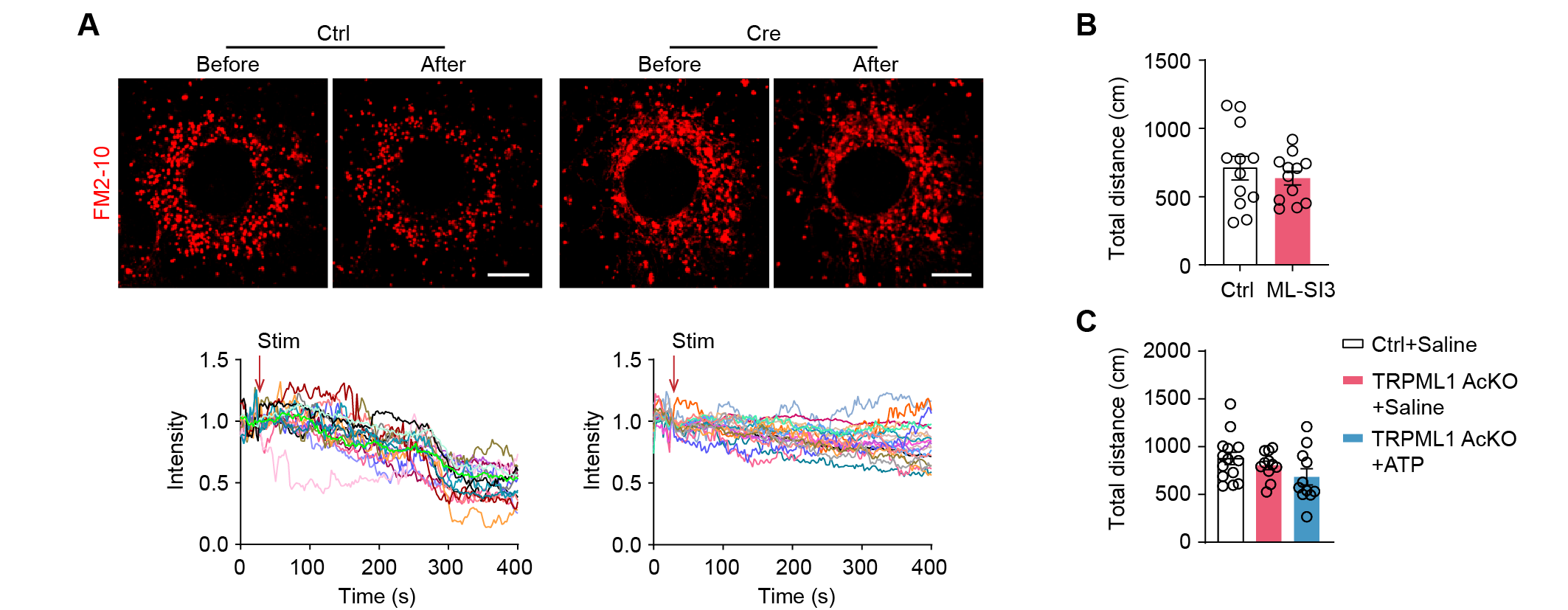
Figure S6. TRPML1 deficiency in astrocytes induces dysfunction of lysosomal exocytosis.** (A) Glutamate-induced changes in fluorescence intensity of FM 2-10 puncta (n = 15-20 puncta per cell). Scale bars, 10 μm. (B) Behavioral performances of C57BL/6J mice in the open field test (OFT) after infusion of ML-SI3 into the mPFC (n = 12). (C) Behavioral performances of TRPML1 AcKO mice treated with ATP in the OFT (n = 11-14). Two-tailed unpaired Student’s t test (B); one way ANOVA followed by Tukey's multiple comparisons test (C). All data are presented as the mean ± SEM.

**
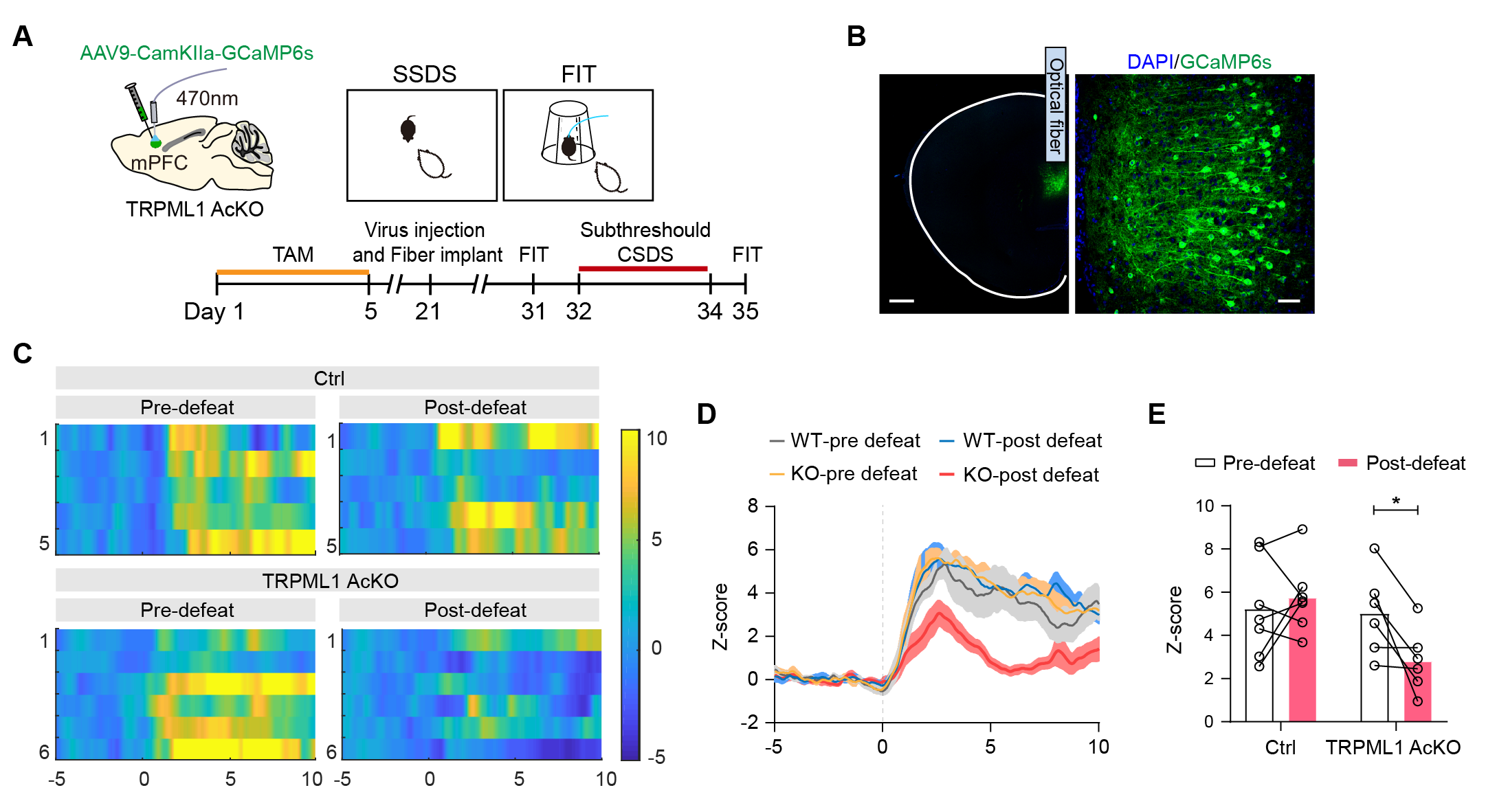
****Figure S7.** **Deletion of astrocytic *Mcoln1* causes deficits in neuronal** **calcium activity in the mPFC under stress.** (A) Schematic of the AAV vectors engineered to express the calcium sensor and design of the fiber photometry experiment. (B) Representative images of calcium sensor expression in mPFC neurons. Scale bars = 500 μm (left), 50 μm (right). (C) Representative heat maps of z-score changes over all trials in individual mice. (D-E) Time course of the average calcium transient z-score event in the forced interaction test (FIT) (D) and quantification of the average peak z score during the FIT (E) (n = 6-7, *P* = 0.0409). Two-way ANOVA followed by Sidak's multiple comparisons test (E). All data are presented as the mean ± SEM. **P* < 0.05.


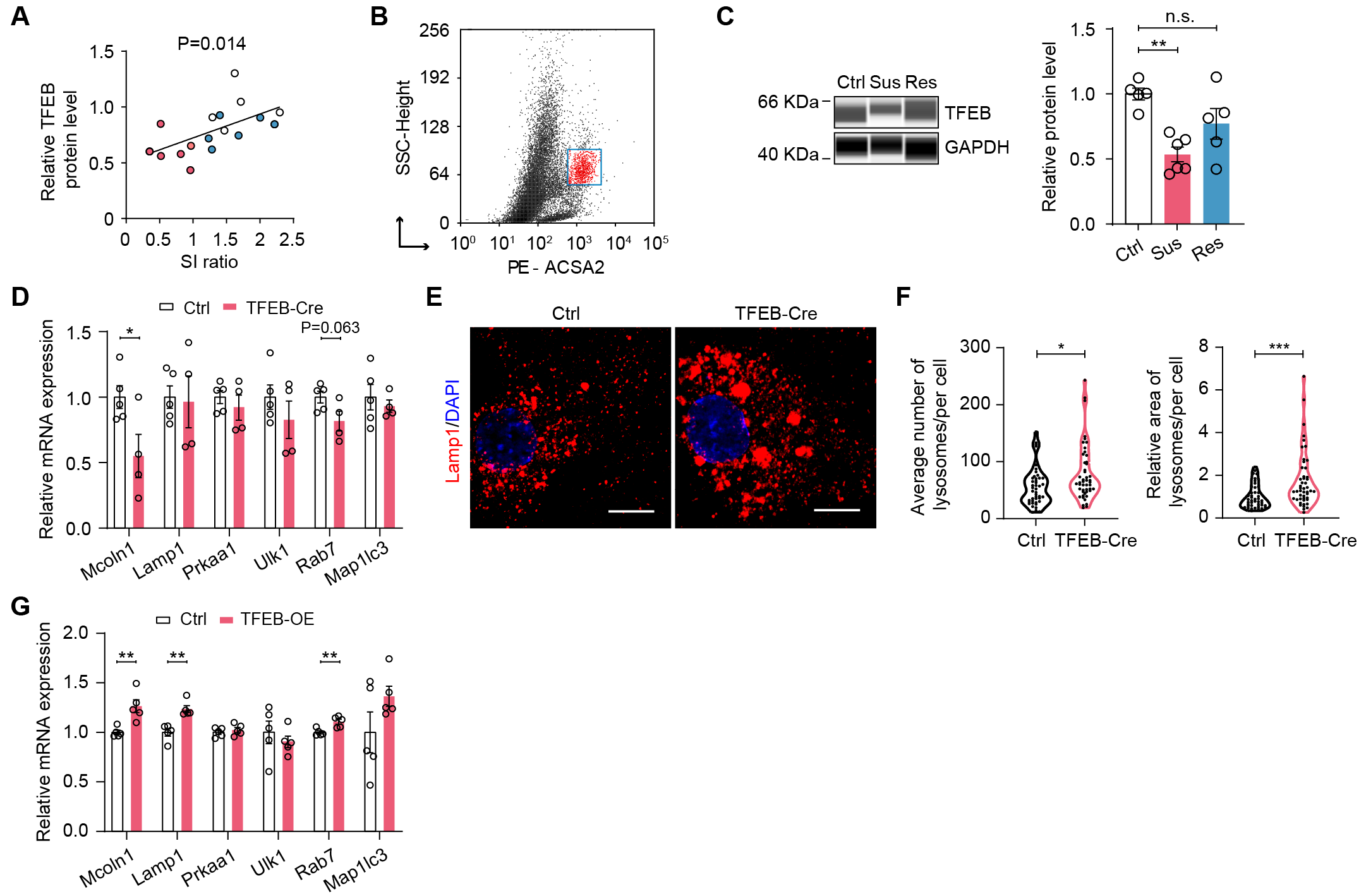


Figure S8. The expression of astrocytic TFEB is attenuated in the mPFC of Sus mice. (A) Correlation between TFEB protein levels and the SI ratio. *P* = 0.014. (B) Analysis of astrocytes isolated by FACS from the mPFC of C57BL/6J mice following the CSDS paradigm. (C) Simple Western blot analysis of TFEB protein levels in mPFC astrocytes of C57BL/6J mice after the CSDS paradigm (n = 5-6, *P* = 0.0016). (D) mRNA levels of several target genes bearing the CLEAR motif in *Tfeb^flox/flox^* astrocytes infected with the Cre or Ctrl virus (n = 4-5, *P* = 0.0373). (E) Representative images of Lamp1 immunostaining in *Tfeb^flox/flox^* astrocytes infected with the Cre or Ctrl virus. Lamp1, red; DAPI, blue. Scale bars, 10 μm. (F) Quantification of lysosomal number and size in TFEB knockdown astrocytes (n = 44-48 cells, *P* = 0.0112 and *P* = 0.0004). (G) mRNA levels of several target genes bearing the CLEAR motif in condition of TFEB overexpression (n = 5, *P* = 0.0051, *P* = 0.0018 and *P* = 0.0059). Correlations evaluated with the Pearson correlation coefficient (A); one way ANOVA followed by Dunnett’s post-hot test (C); two-tailed unpaired Student’s t test (D and G) and Mann Whitney test (F). All data are presented as the mean ± SEM. n.s., not significant; **P* < 0.05.***P* < 0.01, ****P* < 0.001.


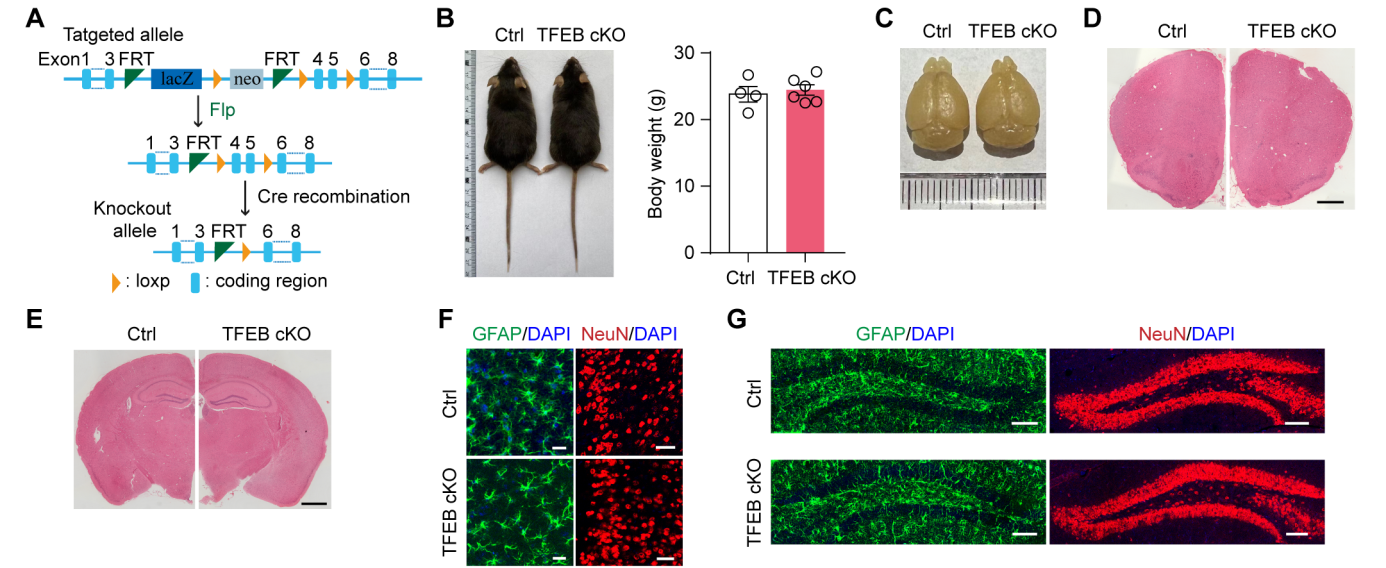


Figure S9. Generation of *Aldh1l1-CreER^T2+/-^; Tfeb^flox/flox^* mice. (A) Schematic of the *Tfeb^flox/flox^* allele. (B) Body weight of TFEB cKO and littermate control mice after TAM injection (n = 4-6). (C) Representative image of the brain size of TFEB cKO and littermate control mice after TAM injection. (D-E) H&E staining of the mPFC (D) and hippocampus (E) from TFEB cKO and Ctrl mice. Scale bars, 1000 µm. (F-G) Immunofluorescence staining of GFAP (green) and NeuN (red) in the mPFC (F) and hippocampus (G) of TFEB cKO and Ctrl mice. Astrocytes were stained with GFAP and DAPI; neurons were stained with NeuN and DAPI. Scale bars = 50 μm (F); 100 μm (G). Two-tailed unpaired Student’s t test (B). All data are presented as the mean ± SEM.


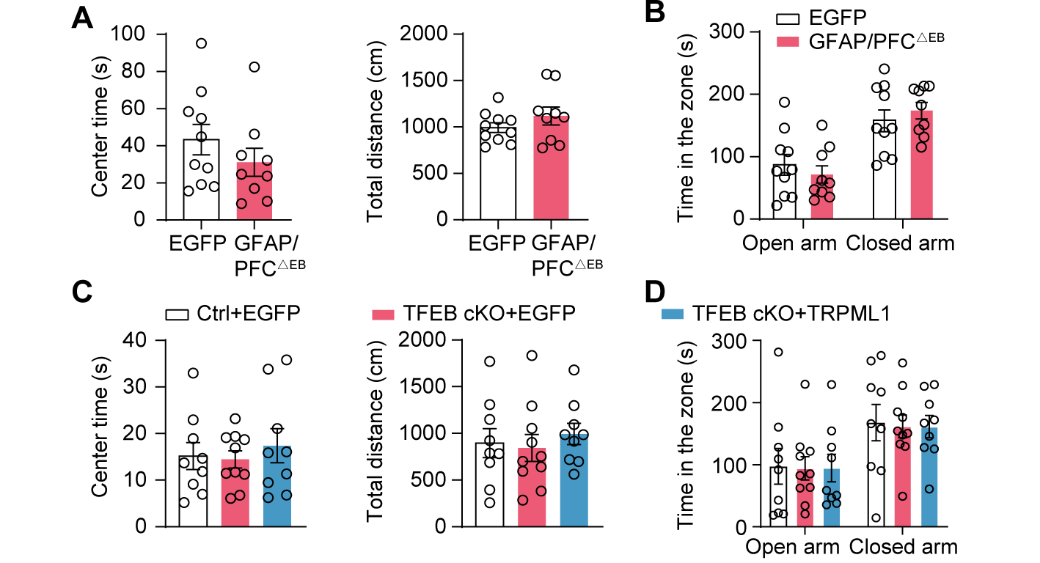


Figure S10. Behavioral performances of GFAP/PFC^△EB^, TFEB cKO, TFEB cKO + TRPML1 and Ctrl mice. (A-B) Behavioral performances of GFAP/PFC^△EB^ and Ctrl mice in the open field test (OFT) and elevated plus maze test (EPM) (n = 9-10). (C-D) Behavioral performances of TFEB cKO + TRPML1, TFEB cKO + EGFP and Ctrl + EGFP mice in the OFT and EPM (n = 9-10). Two-tailed unpaired Student’s t test (A and B); one way ANOVA followed by Tukey's multiple comparisons test (C and D). All data are presented as the mean ± SEM.

Table S1.

Primer sequences used for qRT-PCRs.

| Gene | Primer sequences |
| --- | --- |
| Mcoln1-Forward | 5’-CTGACCCCCAATCCTGGGTAT-3’ |
| Mcoln1-Reverse | 5’-GGCCCGGAACTTGTCACAT-3’ |
| Tfeb-Forward | 5’-CCACCCCAGCCATCAACAC-3’ |
| Tfeb-Reverse | 5’-CAGACAGATACTCCCGAACCTT-3’ |
| Lamp1-Forward | 5’-CAGCACTCTTTGAGGTGAAAAAC-3’ |
| Lamp1-Reverse | 5’-ACGATCTGAGAACCATTCGCA-3’ |
| Prkaa1-Forward | 5’-TGTTCCAGCAGATCCTTTCC-3’ |
| Prkaa1-Reverse | 5’-ATAATTGGGTGAGCCACAGC-3’ |
| Prkaa2-Forward | 5’-GGGTGAAGATCGGACACTACGT-3’ |
| Prkaa2-Reverse | 5’-AAAAGTCTGTCGGAGTGCTGA-3’ |
| Map1lc3a-Forward | 5’-GACCGCTGTAAGGAGGTGC-3’ |
| Map1lc3a-Reverse | 5’-CTTGACCAACTCGCTCATGTTA-3’ |
| Ulk1-Forward | 5’-TTACCAGCGCATCGAGCA-3’ |
| Ukl1-Reverse | 5’-TGGGGAGAAGGTGTGTAGGG-3’ |
| Mtor-Forward | 5’-TCATCAAACAAGCGACATCC-3’ |
| Mtor-Reverse | 5’-GGGCCTCCAGTTACCAGAA-3’ |
| Tlr9-Forward | 5’-GGGCCCATTGTGATGAACC-3’ |
| Tlr9-Reverse | 5’-GCTGCCACACTTCACACCAT-3’ |
| P2rx4-Forward | 5’-ACAACGTGTCTCCTGGCTACAAT-3’ |
| P2rx4-Reverse | 5’-GTCAAACTTGCCAGCCTTTCC-3’ |
| Lgals3-Forward | 5’-TGCCCTATGACCTGCCCTT-3’ |
| Lgals3-Reverse | 5’-TCCTGCTTCGTGTTACACACAA-3’ |
| Lgals9-Forward | 5’-ATGCCCTTTGAGCTTTGCTTC-3’ |
| Lgals9-Reverse | 5’-AACTGGACTGGCTGAGAGAAC-3’ |
| Rab7-Forward | 5’-AGGCTTGGTGCTACAGGAAAA-3’ |
| Rab7-Reverse | 5’-CTTGGCCCGGTCATTCTTGT-3’ |
| Atg4c-Forward | 5’-AGATGAAAGCAAGATGTTGCCT-3’ |
| Atg4c-Reverse | 5’-CCCTGTAGGTCAGCCATATTCTA-3’ |
| Atg2b-Forward | 5’-GTCCCCTTGGACAAATGGTGT-3’ |
| Atg2b-Reverse | 5’-GGACGGACAGGGAAATGGA-3’ |
| Atg16l2-Forward | 5’-GCAGCTTGTGCAGCGTAAG-3’ |
| Atg16l2-Reverse | 5’-CTGGTTGGCCCTCTCTCTAC-3’ |
| Atg6v1g2-Forward | 5’-GAGGAGGCTCAAATGGAGGTG-3’ |
| Atg6v1g2-Reverse | 5’-CTGAACCTGCCGTCTTGTG-3’ |
| Npc1-Forward | 5’-TGTTTGGTATGGAGAGTGTGGA-3’ |
| Npc1-Reverse | 5’-GTCACAGCAGAGACTGACATTG-3’ |
| Gapdh-Forward | 5’-CAATGTGTCCGTCGTGGATCT-3’ |
| Gapdh-Reverse | 5’-GTCCTCAGTGTAGCCCAAGATG-3’ |
| MCOLN1-Forward (human) | 5’-GCTCGTCACCAGCGATGTG-3’ |
| MCOLN1-Reverse (human) | 5’-CTCGATGCCGATCTTCATGA-3’ |
| LGALS3-Forward (human) | 5’-ATGGCAGACAATTTTTCGCTCC-3’ |
| LGALS3-Reverse (human) | 5’-GCCTGTCCAGGATAAGCCC-3’ |
| GAPDH-Forward (human) | 5’-CGGAGTCAACGGATTTGGTC-3’ |
| GAPDH- Reverse (human) | 5’-TGGGTGGAATCATATTGGAACAT-3’ |
| LAMP1-Forward (human) | 5’-CAGCACTCTTTGAGGTGAAAAAC-3’ |
| LAMP1-Reverse (human) | 5’-ACGATCTGAGAACCATTCGCA-3’ |
| MAP1LC3A-Forward (human) | 5’-GACCGCTGTAAGGAGGTGC-3’ |
| MAP1LC3A-Reverse (human) | 5’-CTTGACCAACTCGCTCATGTTA-3’ |
| ULK1-Forward (human) | 5’-TTACCAGCGCATCGAGCA-3’ |
| ULK1-Reverse (human) | 5’-TGGGGAGAAGGTGTGTAGGG-3’ |
| RAB7A-Forward (human) | 5’-AGGCTTGGTGCTACAGGAAAA-3’ |
| RAB7A-Reverse (human) | 5’-CTTGGCCCGGTCATTCTTGT-3’ |
| PRKAA1-Forward (human) | 5’-TGTTCCAGCAGATCCTTTCC-3’ |
| PRKAA1-Reverse (human) | 5’-ATAATTGGGTGAGCCACAGC-3’ |
| Mcoln1-Forward (ChIP-PCR) | 5’-AAGAGGAAGACAGCGTTGAG-3’ |
| Mcoln1-Reverse (ChIP-PCR) | 5’-ATGGGCTGACTCTGAGTTTG-3’ |

Table S2.

Antibodies used for experiments

| Antibodies | Source | Identifier |
| --- | --- | --- |
| Rabbit anti-TRPML1 | Abcam | Cat#ab28508; RRID:AB_776556 |
| Rabbit anti-TFEB | Bethyl | Cat#A303-673A; RRID:AB_11204751 |
| Rabbit anti-TFEB | Cell Signaling | Cat#37785S; RRID:AB_2799119 [PMID:38181789](https://rrid.site/resources/38181789?rpKey=on) |
| Rabbit anti-Phospho TFEB (S211) | Cell Signaling | Cat#37681S; RRID:AB_2799117 |
| Mouse anti-Lamp1 | Abcam | Cat#ab25630; RRID:AB_470708 |
| Rabbit anti-Lamp1 | Abcam | Cat#ab24170; RRID:AB_775978 |
| Rabbit anti-Rab7 | Abcam | Cat#ab137029; RRID:AB_2629474 |
| Goat anti-Iba1 | Novus | Cat#NB100-1028; RRID:AB_521594 |
| Mouse anti-NueN | Cell Signaling | Cat#24307S; RRID:AB_2651140 |
| Mouse anti-GFAP | Cell Signaling | Cat#3670S;RRID: AB_561049 |
| Mouse anti-S100β | Abcam | Cat#ab52642; RRID:AB_882426 |
| Guinea pig anti-S100β | Synaptic Systems | Cat#287004; RRID:AB_2620025 |
| Mouse anti-GAPDH | Proteintech | Cat#60004;  RRID:AB_2107436 |
| Goat anti-LaminB | Santa Cruz Biotechnology | Cat#sc-6217; RRID:AB_648158 |
